# Reshaping immune cell distribution with mRNA noncationic lipid nanoparticles for overcoming neoadjuvant chemo-immunotherapy resistance

**DOI:** 10.7150/thno.136879

**Published:** 2026-07-22

**Authors:** Yiwen Liu, Rui Chang, Kenan Chen, Lin Li, Xiaogang An, Shegan Gao, Dingjun Zha, Hongzhang Deng

**Affiliations:** 1 Department of Otolaryngology-Head and Neck Surgery, Xijing Hospital, Air Force Medical University, Xi’an, Shaanxi 710032, China; 2 Engineering Research Center of Molecular & Neuroimaging Ministry of Education, School of Life Science and Technology, Xidian University, Xi’an, Shaanxi, 710126, China.; 3 Anyang Tumor Hospital, Anyang, Henan, China.; 4 College of Clinical Medicine, The First Affiliated Hospital, Henan University of Science and Technology, Luoyang 471003, Henan, China.

**Keywords:** neoadjuvant chemo-immunotherapy resistance, immune cell distribution, mRNA delivery, macrophage, esophageal squamous cell carcinoma

## Abstract

**Rationale:**

Fusobacterium nucleatum (Fn) is associated with resistance to neoadjuvant chemo-immunotherapy in esophageal squamous cell carcinoma (ESCC), but the underlying mechanism is unclear. We identified Fn-induced SPP1⁺ macrophages as key drivers of a cancer-associated fibroblast (CAF)-mediated spatial immune barrier that restricts CD8⁺ T-cell infiltration.

**Methods:**

Mannose-modified non-cationic thiourea lipid nanoparticles (NC-TNP^M^) were engineered to deliver Cas9 mRNA and SPP1-targeting sgRNA to macrophages. Their therapeutic efficacy was evaluated in Fn-associated ESCC models combined with chemotherapy and anti-PD-L1 treatment.

**Results:**

NC-TNP^M^ achieved efficient SPP1 silencing, markedly reduced SPP1⁺ macrophages, disrupted the macrophage-CAF immune barrier, and restored intratumoral CD8⁺ T-cell infiltration. Combined with chemo-immunotherapy, NC-TNP^M^ significantly suppressed tumor growth, enhanced cytotoxic T-cell activity, promoted macrophage repolarization, and showed no evident toxicity.

**Conclusions:**

Fn-induced SPP1⁺ macrophages drive immune exclusion and chemo-immunotherapy resistance in ESCC. Macrophage-targeted SPP1 editing with NC-TNP^M^ overcomes this barrier and enhances therapeutic efficacy, highlighting a promising nanomedicine strategy for ESCC.

## Introduction

Esophageal squamous cell carcinoma (ESCC) represents a global health burden with particularly high incidence and mortality in Eastern Asia 1-4. While neoadjuvant chemo-immunotherapy remains a cornerstone treatment, therapeutic responses exhibit marked nondeterminacy even among patients with comparable molecular profiles 5. Fn colonization has emerged as a predictor of neoadjuvant chemo-immunotherapy resistance 6, 7, yet an intriguing clinical finding reveals starkly divergent treatment responses among Fn-positive patients despite identical molecular subtypes and otherwise comparable clinicopathological profiles 7-11. Despite the clinical success of neoadjuvant chemo-immunotherapy in locally advanced ESCC, a substantial proportion of patients exhibit primary resistance, and even among those with similar clinicopathological features, treatment responses vary widely 12. Current response stratification relies largely on conventional metrics that fail to capture the underlying biological determinants of resistance. Recent studies have highlighted the tumor immune microenvironment – particularly the spatial organization of immune cells – as a critical modulator of therapeutic efficacy. However, the mechanistic links between intratumoral microbiota, macrophage polarization, spatial immune architecture, and neoadjuvant treatment resistance remain poorly understood. Critically, the mechanistic underpinnings linking intratumoral bacteria to therapeutic failure with neoadjuvant chemo-immunotherapy resistance remain elusive, hindering the development of targeted interventions. Consequently, it is imperative to elucidate the fundamental molecular mechanisms underlying differential neoadjuvant therapy responses in ESCC and their precise interplay with intratumoral Fn. Only through such mechanistic decoding can we develop rational therapeutic strategies to overcome current treatment bottlenecks.

Conventional ESCC treatment stratification relies primarily on clinicopathological parameters such as tumor stage, histologic grade, and PD-L1 expression. However, these metrics often fail to predict individual patient responses, particularly in the setting of neoadjuvant chemo-immunotherapy. Emerging evidence supports a paradigm shift toward biology-guided perioperative decision-making, wherein molecular and immunological features – rather than traditional staging alone–inform treatment selection 13. In this context, understanding how intratumoral microbiota and immune spatial organization govern therapeutic resistance represents a critical step toward personalized ESCC management.

To address this knowledge gap, we pursued an in-depth investigation of the underlying molecular mechanisms of neoadjuvant chemo-immunotherapy resistance. Notably, existing studies on ESCC treatment resistance have exclusively focused on quantifying immune cell abundance within tumors while largely overlooking their spatial distribution. We thus hypothesize that divergent therapeutic outcomes among molecularly homogeneous patients with comparable pre-therapeutic workups primarily stem from differential spatial organization of immune cells. Recent advances highlight tumor microenvironment spatial architecture as a key determinant of treatment efficacy 14-17. Traditional biomarkers focusing on immune cell quantity have failed to explain differential responses, suggesting functional alterations in immune cell distribution may govern neoadjuvant chemo-immunotherapy resistance. Previous study identified that the hepatocellular carcinoma immune barrier, a spatially organized microdomain composed of (CAFs) assembled at the tumor-stroma interface, structurally improves neoadjuvant chemo-immunotherapy resistance and compromises immune checkpoint blockade efficacy through physical exclusion of cytotoxic lymphocytes 18-22. While some literatures reported that SPP1⁺ macrophage accumulation correlates with neoadjuvant failure in ESCC, its spatiotemporal coordination with Fn-driven immunosuppression and resultant chemoresistance constitutes a persistent mechanistic blind spot 23-26.

Addressing dual challenges-clinical (Fn-based prediction inaccuracy) and mechanistic (obscure neoadjuvant chemo-immunotherapy resistance drivers), this study decodes the spatial immune barrier and pioneers a nanotherapeutic strategy that synergistically overcomes ESCC neoadjuvant chemo-immunotherapy resistance. Here, we resolve this mechanistic gap by demonstrating that Fn invades macrophages to drive SPP1 overexpression, triggering fibroblast-dependent assembly of a spatial immune barrier. This physical exclusion zone impedes cytotoxic immune cell infiltration and proliferation, providing a potential explanation for treatment heterogeneity. Building on this discovery, we pioneer a spatial CRISPR/Cas9 genome editing strategy targeting the Fn/SPP1⁺ macrophage/immune distribution axis in ESCC. Mannose-modified non-cationic thiourea lipid nanoparticles (NC-TNP^M^) were prepared to deliver Cas9 mRNA and SPP1 sgRNA for specifically silencing SPP1 in tumor-associated macrophages. Mannose-modified nanoparticles have been reported in previous studies to target macrophages 27, 28. Flow cytometry reveals 92% SPP1 knockdown efficiency in F4/80⁺ macrophages. This nanoplatform overcomes physical immunosuppression by spatially reprogramming lymphocyte topodynamics, boosting chemo-immunotherapy efficacy.

## Results

### The phenomenon of neoadjuvant chemo-immunotherapy resistance

**Figure 1:**
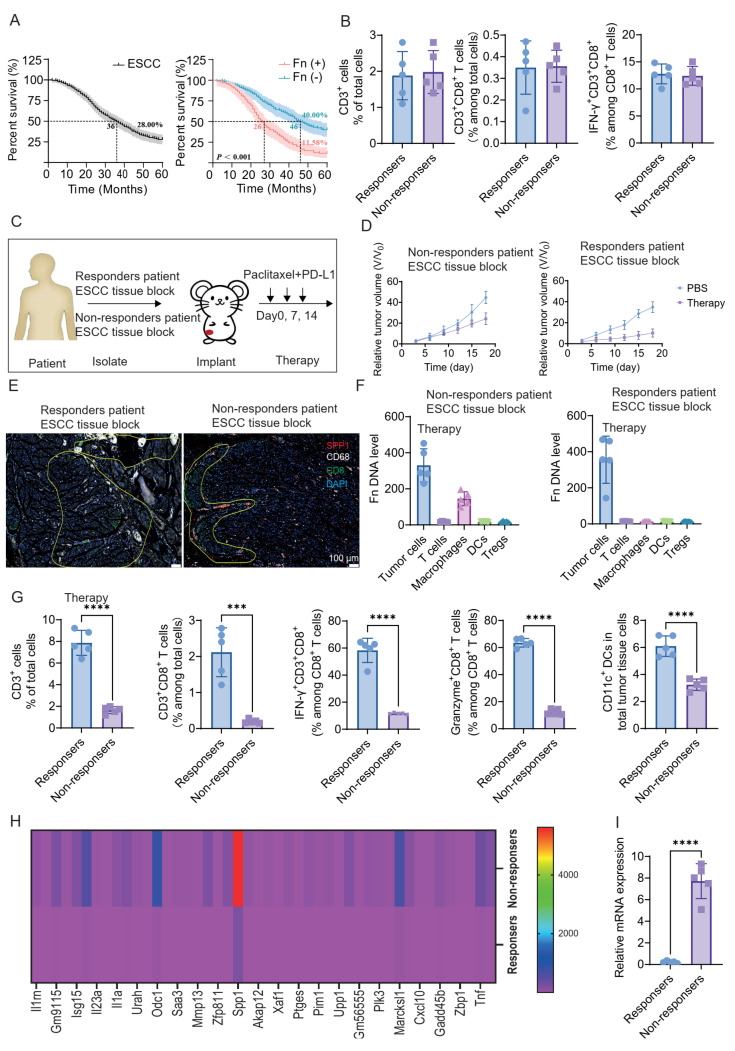
The phenomenon of neoadjuvant chemo-immunotherapy resistance. (A) Fn-positive patients exhibited significantly lower 5-year survival versus Fn-negative cohorts. Among Fn-positive individuals, non-responders to neoadjuvant chemo-immunotherapy showed substantially reduced survival compared to responders. (B) Tumor tissues from Fn⁺ chemo-immunotherapy responder and non-responder patients were dissociated into single-cell suspensions using. After red blood cell lysis and viability staining, cells were stained with fluorochrome-conjugated antibodies against CD45, CD3, CD8, and IFN-γ. Flow cytometry was performed by analyzing ≥ 10,000 live total cells events (mean ± SD; n = 5). (C) Fn⁺ patient-derived chemo-immunotherapy responder and non-responder tumor fragments were engrafted into mice to establish PDX models. On day 0 post-engraftment, cohorts received intravenous tail vein injections of either PBS (control), therapeutic agents, or anti-PD-L1 antibody on days 0, 7, and 14. (D) Relative tumor volume curves following treatment. Data normalized to baseline volume (Day 0). (E) Immunofluorescence analysis of resected tumor sections from the non-responders and responders groups with therapy treatment. (F) Fn DNA copies were quantified by qPCR in FACS-sorted cells (purity > 97%) (mean ± SD; n = 5). (G) Flow cytometry analysis of immune cell populations in single-cell suspensions from resected post-treatment tumors from therapy groups (mean ± SD; n = 5). (H) Comparative transcriptomics analysis of responder and non-responder tumors with therapy treatment. (I) RT-qPCR analysis of SPP1 in CD68⁺ macrophages isolated from responder vs. non-responder post-treatment tumors (mean ± SD; n = 5).

Clinical management of ESCC is frequently challenged by intrinsic chemoresistance and suboptimal treatment outcomes 29, 30. The integration of neoadjuvant chemotherapy with PD-L1 blockade has suggested superior efficacy; however, tumor-resident Fn has emerged as a putative biomarker for treatment response 31-33. Conventional clinical wisdom posits that Fn-positive status correlates with inferior outcomes, while Fn-negative patients exhibit favorable responses. The detail information of Clinical samples was shown in Supporting EXCEL and the inclusion and exclusion criteria are shown in Figure S1. Our analysis of 5-year survival rates under combination therapy revealed: Fn-positive patients suggested 70% lower overall survival compared to Fn-negative counterparts (Figure 1A). Integrating pathological response assessment with survival outcomes revealed critical dissociations: within the Fn-positive cohort, the 20% achieving exceptional long-term survival suggested significantly enhanced treatment efficacy. Conversely, among Fn-negative patients, the 30% with suboptimal survival exhibited markedly diminished therapeutic response (Figure S2). Among Fn-positive patients, a subset responded well to neoadjuvant chemo-immunotherapy, while the majority exhibited poor treatment efficacy with significantly lower 5-year survival rates. Conversely, within the Fn-negative cohort, a subset displayed treatment resistance and suboptimal outcomes, though the majority achieved favorable therapeutic responses and suggested notably higher 5-year survival rates compared to Fn-positive patients. Therefore, we preliminarily propose that the association between Fn and resistance to neoadjuvant chemo-immunotherapy in ESCC represents not merely a superficial correlation, but rather reflects a profound underlying causal link. Fn appears to be a mechanistic driver mediating this therapeutic resistance. Interrogating the immunological basis of differential treatment responses in Fn⁺ ESCC, we discovered that conventional immune metrics, including cytotoxic lymphocyte density, failed to distinguish responders from non-responders, challenging quantity-centric resistance paradigms (Figure 1B). To investigate tumor-intrinsic determinants of treatment response, we suggested patient-derived xenograft (PDX) models using tumor tissues from ESCC patients stratified by neoadjuvant chemo-immunotherapy outcomes: responders and non-responders. Following subcutaneous implantation, mice received intravenous combination therapy (chemotherapy + anti-PD-L1) on days 0, 7, and 14 (Figure 1C). Tumor growth curves suggested significantly superior suppression in PDXs derived from clinical responders versus non-responders (Figure 1D). Using the same PDX model system, we sacrificed mice at day 7 post-treatment initiation and performed multiplex immunofluorescence staining on tumor sections. The results revealed comparable overall immune cell densities between responder-derived and non-responder-derived groups (Figure 1E). However, profound differences in T-cell spatial distribution were observed. In the responders group, T cells exhibited diffuse infiltration patterns with penetration throughout tumor nests (mean infiltration depth: 128 ± 18 μm). For the non-responders group, T cells were spatially confined within macrophage/Fn-enriched stromal compartments, demonstrating physical exclusion from tumor cores. Those results confirmed T-cell sequestration within Fn-dense peripheral in non-responders group specimens. Subsequent analysis of cell type-specific Fn colonization revealed profoundly elevated bacterial burdens in macrophages from non-responders group tumors. Quantification suggested that for pre-treatment, non-responders group macrophages harbored 185 Fn copies/μg DNA in macrophage vs 13.65 in the responders group (p < 0.0001), for post-treatment: the non-responders group maintained 148.6 ± 22.1 Fn copies/μg DNA vs 0.9 ± 0.2 in the responders group (p < 0.0001). This represents ≈ 150-fold higher Fn colonization in non-responders across therapeutic intervention (Figure 1F and Figure S3). We therefore hypothesize that macrophage phenotypic reprogramming rather than Fn constitutes a key driver of chemo-immunotherapy resistance in ESCC, with Fn serving merely as a contributing factor. To test this hypothesis, we first compared post-treatment immune cell densities between the responders and non-responders groups. Following treatment, samples were taken from the inner regions of the tumor tissue and subjected to lysis. A clear difference in immune cell infiltration could be observed. In the untreated groups, however, there was little difference in immune cell infiltration between tumors derived from treatment-sensitive patients and those from treatment-insensitive patients (Figure 1G and Figure S4). Using magnetic-activated cell sorting (MACS), we isolated tumor-associated macrophages (TAMs) from ESCC tissues of different response groups. Transcriptomic analyses revealed profound SPP1 overexpression in TAMs of the non-responders group, demonstrating 20-fold higher expression levels compared to the responders group (Figure 1H and Figure S5). RT-qPCR analysis revealed that TAMs from PDX models derived from poor-response ESCC patients exhibited 30.86-fold higher SPP1 gene expression compared to those from response group (Figure 1I). Collectively, our findings establish SPP1 overexpression in tumor-associated macrophages as a key determinant of neoadjuvant chemo-immunotherapy resistance in ECSS. However, the molecular underpinnings driving this pathogenic overexpression remain to be elucidated. Crucially, definitive validation is required to determine whether Fn colonization serves as the fundamental inducer of SPP1 hyperexpression in the tumor microenvironment.

### The mechanism and therapy method

**Figure 2:**
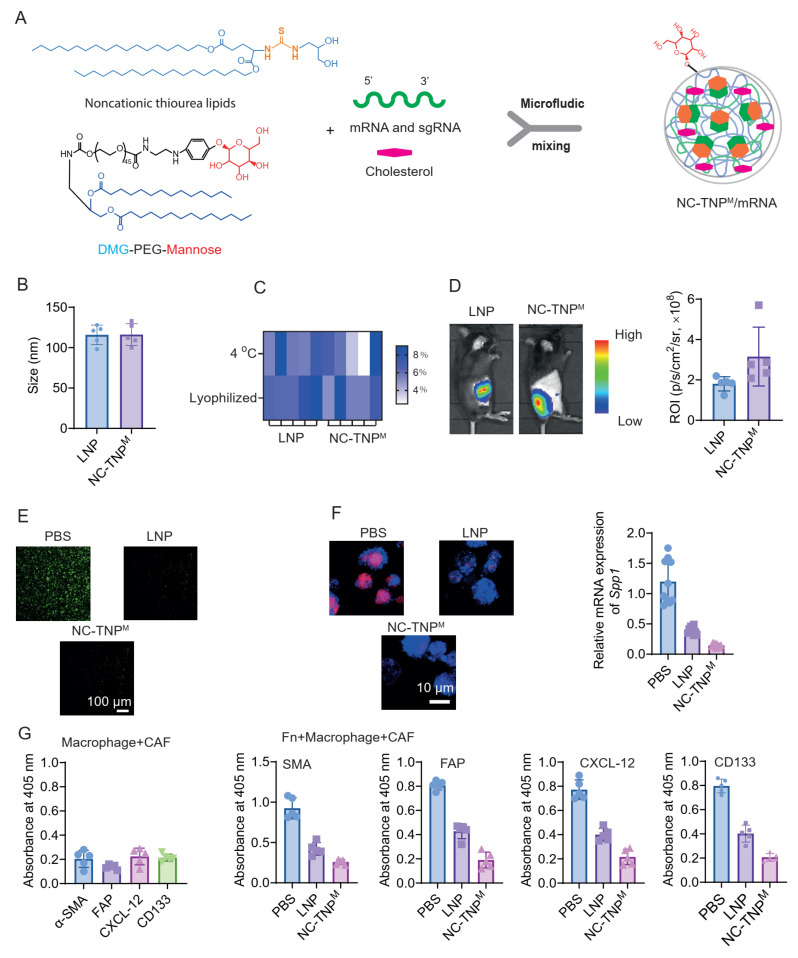
Design and characterization of delivery system. (A) Microfluidic mixing of noncationic thiourea lipids and DMG-PEG mannose (ethanol phase), cholesterol and mRNA/sgRNA (aqueous phase) forming stable core-shell nanoparticles through multivalent hydrogen bonding. (B) The size distributions of NC-TNP^M^. (C) mRNA structural integrity in LNP- and NC-TNP^M^-encapsulated formulations in the state of liquid phase and lyophilized powder following 30-day storage at 4°C: Comparative analysis of liquid versus lyophilized states. (D) *In vivo* bioluminescence imaging (left) and quantitative analysis result (right) of C57BL/6 mice 48 hours post-intravenous administration of LNP-Luc mRNA versus NC-TNP^M^-Luc mRNA (0.5 mg/kg luciferase-encoding mRNA). (E) CRISPR-mediated GFP disruption in engineered RAW264.7-GFP cells treated with LNP or NC-TNP^M^ (Cas9 mRNA and SPP1-targeting gRNAs). Gene editing efficacy was quantified by confocal microscopy based on fluorescence loss. Scale bars: 100 µm. (F) Macrophages were first co-cultured with F. nucleatum (MOI 50:1, 24h) to establish SPP1⁺ phenotype. Post-NC-TNP^M^ treatment, SPP1 downregulation efficiency was quantified by immunofluorescence staining (left), and RT-qPCR (right). (G) CAF-associated biomarkers (SMA, FAP, CXCL12, and CD133) were quantified by ELISA in two experimental models: macrophages alone and Fn-infected macrophages co-cultured with cancer-associated fibroblasts, results presented as optical density at 450 nm (OD₄₅₀, mean ± SD, n = 5).

Although this study has not fully elucidated the detailed mechanisms underlying neoadjuvant chemo-immunotherapy resistance in esophageal squamous cell carcinoma, we have suggested its association with macrophage-specific SPP1 overexpression. We therefore investigated whether Fn serves as a key inducer of this pathogenic SPP1 hyperexpression. *In vitro* infection assays using macrophage cell lines suggested robust intracellular colonization of Fn within macrophages (Figure S6). Significantly, Fn infection dramatically upregulated SPP1 expression (> 8-fold increase vs. uninfected controls), establishing direct bacterial induction of this resistance biomarker (Figure S7). We suggest a pathogenic cascade wherein Fn intracellular colonization reprograms macrophages via SPP1 hyperexpression, creating a permissive microenvironment for chemo-immunotherapy resistance in ESCC through unidentified effector mechanisms. To elucidate the underlying mechanistic link between SPP1⁺ macrophages and therapeutic resistance, we will employ targeted SPP1 suppression strategies. This dual-pronged approach simultaneously enables therapeutic development and mechanistic discovery by probing how barrier disruption restores treatment sensitivity. We designed a dual-purpose nanoplatform using mannose-modified non-cationic thiourea lipid nanoparticles (NC-TNP^M^) with macrophage targeting ability to co-deliver Cas9 mRNA and SPP1-targeting sgRNAs. We employed microfluidic synthesis to co-encapsulate Cas9 mRNA and SPP1-targeting sgRNA within NC-TNP^M^ for targeted delivery (Figure 2A). The preparation method adopts the method previously employed in our group 34. This approach simultaneously disrupts macrophage-mediated spatial barriers therapeutically and mechanistically deciphers SPP1's role in immune exclusion through CRISPR-enabled pathway dissection. Using conventional LNP formulations (containing SM-102 [1-octylnonyl 8-((2-hydroxyethyl)(6-oxo-6-(undecyloxy)hexyl)amino)octanoate], DSPC [1,2-distearoyl-sn-glycero-3-phosphocholine], cholesterol, and DMG-PEG2000 [1,2-dimyristoyl-rac-glycero-3-methoxypolyethylene glycol] at 50:10:38.5:1.5 molar ratio) as a control group 35-40, dynamic light scattering (DLS) analysis suggested that NC-TNP^M^ nanoparticles exhibited a hydrodynamic diameter of approximately 100 nm (Figure 2B). We assessed the long-term stability and bioactivity of NC-TNP^M^/mRNA formulations in both liquid and lyophilized states during 30-day storage at 4 °C. Compared to LNP/mRNA controls, NC-TNP^M^/mRNA exhibited superior mRNA integrity preservation (Figure 2C). The NC-TNP^M^ formulation was characterized by encapsulation efficiency (RiboGreen assay), drug loading capacity (mass ratio of mRNA/sgRNA to total lipid), and serum stability (integrity of encapsulated mRNA after incubation in 50% mouse serum at 37 °C over time), as summarized in Table S1. This suggests NC-TNP^M^'s exceptional capacity to preserve mRNA bioactivity during long-term storage. Bioluminescence imaging and quantitative analysis suggested comparable luciferase expression levels, with both LNP and NC-TNP^M^ formulations exhibiting robust *in vivo* transfection efficiency (Figure 2D). We next assessed the genome-editing efficacy of NC-TNP^M^ loaded with Cas9 mRNA and SPP1-targeting sgRNA. GFP-expressing RAW264.7 cells were incubated with NC-TNP^M^ in complete medium for 24 hours, followed by confocal laser scanning microscopy (CLSM) analysis (Figure 2E). Quantitative fluorescence measurements revealed NC-TNP^M^-treated cells exhibited a 95% reduction in signal intensity (p < 0.0001) relative to PBS-treated controls maintaining high GFP expression (Figure S8). Following 24-hour Fn infection that induced SPP1 hyperexpression in macrophages, NC-TNP^M^ nanoparticles loaded with Cas9 mRNA and SPP1-targeting sgRNA were administered. Immunofluorescence staining (Figure 2F, left), RT-PCR analysis (Figure 2F, right) suggested potent SPP1 suppression, achieving approximately 90% reduction in expression compared to PBS-treated controls. Therefore, we can confirm that the macrophage-targeting NC-TNP^M^ system loaded with Cas9 mRNA and SPP1-targeting sgRNA, constructed in this study, effectively reduces SPP1 expression levels in macrophages. This offers a promising solution to overcome resistance to neoadjuvant chemo-immunotherapy in ESCC. Based on the preceding results, Figure 1E indicates that in ESCC, the high expression of SPP1 in macrophages contributes to immunotherapy resistance not by reducing the ability to recruit immune cells, but rather by impeding the infiltration and distribution of the recruited immune cells. In this regard, previous research findings have reported that SPP1-positive macrophages interact with fibroblastic cells to form an immune cell exclusion barrier 19. This barrier hinders the distribution of immune cells and alters their spatial arrangement within the tumor. Consequently, although the total number of immune cells within the tumor may be comparable between the two groups, their therapeutic efficacy differs significantly. Therefore, to investigate the interaction between Fn-infected SPP1-high macrophages and tumor fibroblasts, our co-culture assays suggested that in the control group (untreated macrophages lacking SPP1 expression), co-culture with fibroblasts had no effect on CAF viability. Conversely, Fn-infected SPP1-high macrophages significantly promoted the upregulation of cancer-associated fibroblast markers when co-cultured with CAFs (Figure 2G). We knocked down SPP1 in Fn-infected macrophages using NC-TNPM (Cas9 mRNA + SPP1 sgRNA). SPP1 knockdown completely abolished CAF activation and proliferation in co-culture (Figure S9). These data suggest that macrophage SPP1 overexpression is the key factor driving CAF activation and proliferation.

### The interaction between SPP1-positive macrophages and CAFs

**Figure 3:**
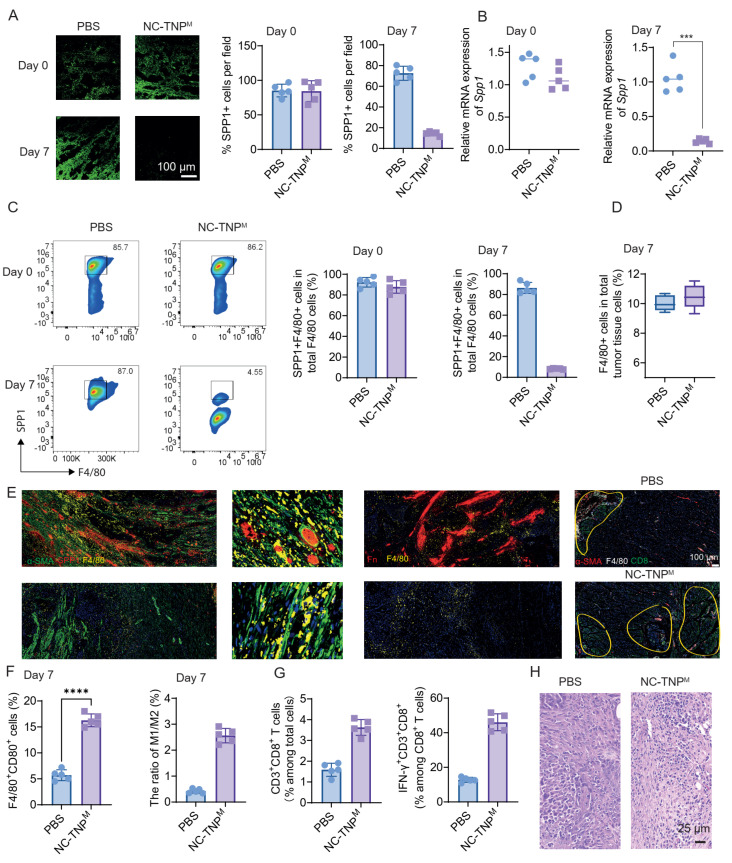
Fn-triggered SPP1^+^ macrophages co-incubated with CAFs to form immune barriers that remodel immune cell spatial distribution. (A) Fn-drug-induced ESCC model in C57BL/6 mice, NC-TNP^M^ loaded with Cas9 mRNA and SPP1-targeting sgRNA were administered via tail vein injection on Day 0. Mice were sacrificed on Day 7, whereafter ESCC tissues were harvested and subjected to immunofluorescence staining (left) quantitative statistical results (right) to detect SPP1 expression. (B) Tumor tissues were collected. On day 0 and day 7, macrophages were isolated via magnetic bead sorting, and SPP1 mRNA expression levels were quantified in these cells using qRT-PCR. (C) Flow cytometric dot plots (left) and quantitative analyses (right) were used to assess SPP1⁺macrophage populations across different treatment groups at baseline (Day 0) and endpoint (Day 7). (D) Quantitative flow cytometry was performed to determine total F4/80⁺ macrophage counts. (E) Representative IHC of ESCC tissues from differentially treated mice showing F. nucleatum burden, SPP1⁺ cell density, F4/80⁺ macrophage infiltration, and T-cell spatial distribution/density. (F) Following intravenous administration of NC-TNP^M^, flow cytometric analysis revealed altered abundance and phenotypic profiles of TAMs within the tumor microenvironment. (G) Quantitative flow cytometric analysis of immune cell infiltration within the tumor parenchyma. (H) H&E (Hematoxylin and Eosin) staining of tumor tissues.

To preliminarily evaluate the biosafety of our system, we performed histological analysis (H&E staining) of major organs following systemic administration of nanoparticles, along with liver function assessments (Figure S10 and 11). The results showed no evident tissue damage or functional impairment, indicating favorable biosafety of the NC-TNPM platform. Following establishment of the Fn-drug induced ESCC model in C57BL/6 mice, NC-TNP^M^ loaded with Cas9 mRNA and SPP1-targeting sgRNA were administered via tail vein injection on Day 0. Mice were sacrificed on Day 7, whereafter ESCC tissues were harvested. SPP1-positive cell density in tumor regions was quantified using immunofluorescence staining. As shown in Figure 3A, mice receiving intravenous NC-TNP^M^ exhibited a significant reduction in SPP1⁺ cell frequency, nearly 85% decrease compared with PBS controls by Day 7. Using MACS, we isolated TAMs from ESCC tissues of different treatments groups. RT-qPCR analysis revealed elevated baseline SPP1 expression in tumor-associated macrophages (Figure 3B). By Day 7 post-treatment, NC-TNP^M^ administration reduced SPP1 transcript levels by 85% compared to pre-treatment values (p < 0.001) (Figure 3B). As shown in Figure 3C, tumor tissues were harvested on Day 7 post-treatment, enzymatically dissociated into single-cell suspensions using collagenase, and subjected to flow cytometry. Analysis revealed that NC-TNP^M^ loaded with Cas9 mRNA and SPP1-targeting sgRNA significantly reduced the proportion of SPP1⁺ macrophages. Specifically, SPP1⁺F4/80⁺ macrophage frequency decreased by 93.5% compared to pre-treatment levels in the NC-TNP^M^ group. To determine whether the observed reduction was specific to the SPP1⁺ subset rather than total macrophage depletion, we quantified F4/80⁺ macrophage abundance pre- and post-treatment. Flow cytometric analysis confirmed comparable total macrophage levels before and after therapy (Figure 3D and Figure S12). These results suggested that our macrophage-targeted delivery system for Cas9 mRNA and SPP1 targeting sgRNA achieves highly efficient reduction of SPP1⁺ macrophages *in vivo*. This targeted approach shows significant potential to overcome resistance to neoadjuvant chemoradiotherapy combined with immunotherapy in ESCC. Combined analysis of Figure 1E, preliminarily indicates that a key mechanism underlying neoadjuvant chemo-immunotherapy resistance in ESCC with high SPP1⁺ macrophage infiltration involves functional interplay between SPP1⁺ macrophages and fibroblasts, which compromises therapeutic efficacy through spatial barrier formation. As shown in Figure 3E, immunohistochemical analysis of excised tumors pre- and post-NC-TNP^M^ treatment revealed conserved Fn distribution and significant T-cell spatial reorganization despite unaltered total T-cell numbers (p = 0.38). Post-treatment tumors exhibited uniformly dispersed T cells with deep parenchymal infiltration, whereas pre-treatment/PBS controls showed T cells spatially constrained in peri-stromal clusters colocalized with SPP1⁺ macrophages and CAFs, indicating NC-TNP-mediated dissolution of physical exclusion barriers. While Fn infection did not alter total macrophage abundance in tumors, it significantly skewed macrophage polarization toward an M2-like phenotype at the expense of M1-like populations. Our engineered NC-TNP^M^ system effectively restored the M1/M2 balance (Figure 3F). Flow cytometric analysis further suggested that NC-TNP^M^ treatment substantially enhanced tumor infiltration of both total CD3⁺CD8⁺ T cells (2.3-fold increase) and IFN-γ-producing cytotoxic T cells (4.3-fold increase), indicating potential to improve immunotherapy efficacy (Figure 3G). H&E staining of tumor tissues further suggested enhanced immune cell infiltration and significantly reduced CAF content post-treatment. H&E staining of tumor tissues further suggested enhanced immune cell infiltration (Figure 3H). Collectively, our results establish that resistance to neoadjuvant chemo-immunotherapy in esophageal squamous cell carcinoma originates from Fn-driven SPP1 upregulation in tumor-associated macrophages. This induces: (1) hyperproliferation of cancer-associated fibroblasts; (2) Formation of SPP1⁺ macrophage-CAF complexes that organize into immune-excluding barriers. These barriers spatially constrain lymphocytes without reducing their abundance, preventing uniform tissue distribution required for effector function. Critically, this architecture also impedes infiltration and expansion of therapy-recruited lymphocytes. Over extended treatment durations, the persistent barrier progressively impairs lymphocyte penetration and clonal expansion, ultimately compromising therapeutic efficacy. To dissect the molecular mechanisms by which Fn-infected SPP1⁺ macrophages activate CAFs, we performed transwell co-culture assays (0.4 μm pore size) using Fn-infected RAW264.7 macrophages and major ESCC-derived CAFs. Cytokine array analysis of conditioned media revealed that co-culture of Fn-infected macrophages with CAFs significantly upregulated the secretion of several pro-fibrotic factors, most notably TGF-β1 (8.5-fold) and IL-6 (6.2-fold), compared with control co-cultures (uninfected macrophages + CAFs) (Figure S13). To further address the spatial heterogeneity of immune responses within the tumor, we performed the following experiment. C57BL/6 mice bearing subcutaneous mouse-derived ESCC tumors were divided into two groups: (1) Fn-treated group, which received intratumoral multipoint injections of Fusobacterium nucleatum (1×10⁸ CFU, twice weekly for 2 weeks); and (2) control group, which received tumor-bearing mice without Fn injection. When tumor size reached approximately 1.2 cm in diameter, mice were euthanized, and tumors were harvested. To assess spatial differences in immune responses, we designed a radial sampling scheme as illustrated in the schematic diagram (Figure S14). Starting from the geometric center of the tumor, we collected serial tissue blocks (2 mm × 2 mm each) along outward radial directions. This approach allowed us to simulate and compare the immune response profiles across different spatial regions, from the tumor core to the periphery.

As shown in Figure S15, the results showed that the proportion of SPP1⁺ macrophages in the Fn-positive group was significantly higher than that in the Fn-negative group. When examining the spatial distribution across the tumor, the immune cell distribution in Fn-negative tissues did not vary dramatically across different regions. In contrast, in Fn-positive tissues, it was clearly evident that immune cell distribution was poorer in the central region of the tumor. These findings suggest, to some extent, that there are spatial differences in immune cell distribution within the tumor tissue. Although multiplex immunofluorescence and immunohistochemistry consistently demonstrated spatial redistribution of immune cells following NC-TNPM treatment, these observations remain largely descriptive. Our proposed model of SPP1⁺ macrophage–CAF-mediated immune exclusion is supported by colocalization and spatial distribution patterns but lacks more rigorous quantitative spatial analyses, such as nearest-neighbor measurements, spatial statistics, or spatial transcriptomics. Moreover, our data do not directly demonstrate that CAFs form a continuous physical barrier to T-cell infiltration. Instead, they show that SPP1⁺ macrophage-driven CAF activation is associated with extracellular matrix remodeling and altered T-cell localization. Thus, we interpret these findings as evidence for a functional stromal barrier rather than definitive proof of physical immune exclusion. Future studies integrating high-resolution spatial transcriptomics, intravital imaging, and functional perturbation will be required to establish the underlying spatial mechanisms.

### NC-TNP^M^ overcome neoadjuvant chemo-immunotherapy resistance in subcutaneous tumor model

We suggested a mouse model bearing ESCC tumors. When the tumors reached approximately 200 mm³, we intravenously injected Cy5-labeled NC-TNPM and monitored its *in vivo* distribution at 4 h, 12 h, 24, and 48 h post-injection.

**Figure 4:**
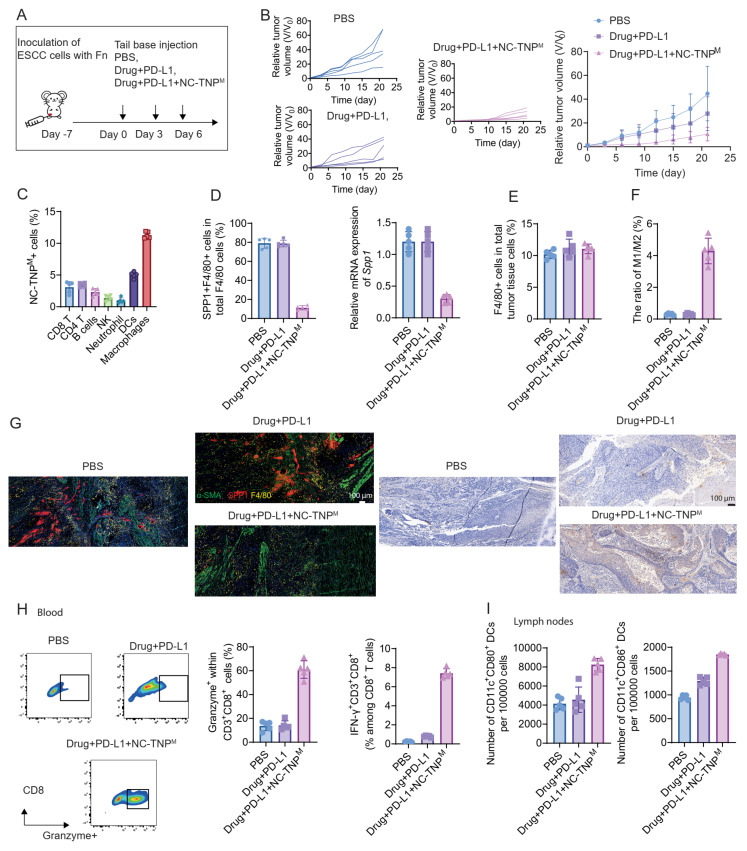
*In vivo* validation of SPP1 knockdown for alleviating resistance to neoadjuvant chemoradiotherapy combined with Immunotherapy. (A) Schematic of experimental timeline: C57BL/6 mice bearing subcutaneous mouse-derived ESCC tumors received Fn (1×10⁸ CFU, multipoint intratumoral, twice weekly × 2 weeks). When tumors reached ≈100 mm³ (Day 0), mice were treated with: PBS control, Chemotherapy + anti-PD-L1 (10 mg/kg), Macrophage-targeted NC-TNP^M^ (5 mg/kg). (Arrows: Days 0/3/6 administration). (B) Relative tumor volume curves following treatment. (C) Quantification of fluorescently labeled NC-TNP^M^ accumulation across major cell populations (mean ± SD; n = 5). (D) Tumors were excised, enzymatically dissociated into single-cell suspensions using collagenase IV (1 mg/mL, 37°C, 45 min), and subjected to MACS with anti-F4/80 microbeads to isolate macrophages. SPP1 expression was subsequently quantified via flow cytometry and RT-qPCR (mean ± SD; n = 5). (E) The ratio of F4/80^+^ cells. (F) M1/M2 ratio quantification across treatment groups. (G) Representative images of *ex vivo* mouse tumor sections stained to visualize the spatial distribution of SPP1⁺ macrophages, α-SMA⁺ fibroblasts, and CD8⁺ T cells within the tumor tissue. (H) Peripheral blood was collected from mice, and peripheral blood mononuclear cells (PBMCs) were isolated for flow cytometry analysis of immune cell populations, including Granzyme⁺CD8⁺ T cells, and IFN-γ⁺CD3⁺CD8⁺ T cells (mean ± SD; n = 5). (I) Sentinel lymph nodes were harvested from mice, dissociated into single-cell suspensions, stained, and analyzed by flow cytometry to assess dendritic cell (DC) maturation (mean ± SD; n = 5).

As shown in Figure S16, Cy5-labeled NC-TNP^M^ exhibited time-dependent accumulation in the tumor, with peak fluorescence observed at 24 h post-injection. At 24 h, the tumor-to-liver ratio was 0.42 and the tumor-to-spleen ratio was 0.38, indicating preferential but not exclusive accumulation in macrophage-rich organs. The mechanism underlying resistance to neoadjuvant chemoradiation and immunotherapy in ESCC is now suggested: Fn invasion of macrophages drives SPP1 overexpression, which triggers hyperproliferation of CAFs and subsequent deposition of fibrous matrix proteins. This SPP1⁺ macrophage-CAF collaboration generates dense, immune-excluding physical barriers that spatially constrain intratumoral immune cell distribution without altering lymphocyte abundance, ultimately compromising therapeutic efficacy by impeding immunocyte functionality. However, the underlying mechanisms determining why only a subset of Fn positive patients exhibit intramacrophage invasion—while others do not—remain beyond the scope of this study. Following subcutaneous implantation of mouse-derived ESCC cells in C57BL/6 mice, animals were randomized into: untreated controls and Fn exposed cohorts (1×10⁸ CFU, multipoint intratumoral, twice weekly ×2 weeks). Fn-exposed tumors suggested elevated SPP1⁺ macrophage infiltration (Figure S17 and S18). At 2 weeks post-Fn initiation, *in vivo* imaging and quantitative statistical analysis of the fluorescence intensity at the tumor site at different time points after intravenous injection of Cy5-labeled NC-TNP^M^. The results showed that the nanoparticles exhibited effective accumulation at the tumor site (Figure S19). The same model mentioned above, Fn-exposed mice received triweekly treatments (Days 0/3/6) of: Chemotherapy (1 mg/kg of cisplatin and 4 mg/kg of paclitaxel were intraperitoneally administered) + anti-PD-L1 (10 mg/kg), Macrophage-targeted NC-TNP^M^ (5 mg/kg for mRNA, 10 mg/kg for sgRNA), Combination therapy, PBS control (Figure 4A). Tumor growth curves suggested that NC-TNP^M^ significantly enhanced therapeutic efficacy, yielding superior tumor inhibition rates (Figure 4B). Analysis of intratumoral cellular targeting revealed preferential accumulation in macrophages (Figure 4C). The results indicate that the delivery system can efficiently target and deliver to macrophages, thereby minimizing off-target side effects. Furthermore, flow cytometry (Figure 4D left) and qRT-PCR (Figure 4D right) analysis of F4/80⁺ macrophages isolated via magnetic sorting from dissociated tumors showed dramatic suppression of SPP1⁺ macrophage frequency in the NC-TNP^M^ group—representing an 85% reduction compared to the chemo/anti-PD-L1 monotherapy group (p < 0.001). Concomitantly, NC-TNP^M^ treatment significantly elevated M1-like polarized macrophage (MHC-II⁺CD80⁺) infiltration within tumors—further demonstrating its capacity to reprogram the immunosuppressive microenvironment while depleting SPP1⁺ macrophages (Figure 4E-F). Immunofluorescence analysis confirmed significantly lower SPP1⁺ macrophage density in NC-TNP^M^-treated tumors versus chemo/anti-PD-L1 monotherapy (p < 0.001) (Figure 4G). Concomitant reduction in CAF abundance and disrupted barrier function were observed, resulting in fundamentally restructured CD8⁺ T-cell spatial distributions: NC-TNP^M^ enabled deep, uniform parenchymal infiltration, while control groups exhibited CD8⁺ T cells physically constrained adjacent to CAFs by persistent macrophage-fibroblast barriers that blocked pan-tumoral dispersion. Peripheral blood mononuclear cells (PBMCs) were isolated via red blood cell lysis and analyzed by flow cytometry. NC-TNP^M^ treatment induced a 5-fold increase in Granzyme⁺CD8⁺ T and IFN-γ⁺CD3⁺CD8⁺ cytotoxic T lymphocytes compared to chemo/anti-PD-L1 monotherapy, confirming potent activation of tumor-specific effector functions in circulating T cells (Figure 4H). Furthermore, NC-TNP^M^ treatment also enhanced DCs maturation and reduced the immunosuppressive cells in the tumor tissue compared to the PBS group (Figure 4I and Figure S20).

### Therapeutic efficacy in the PDX model

**Figure 5:**
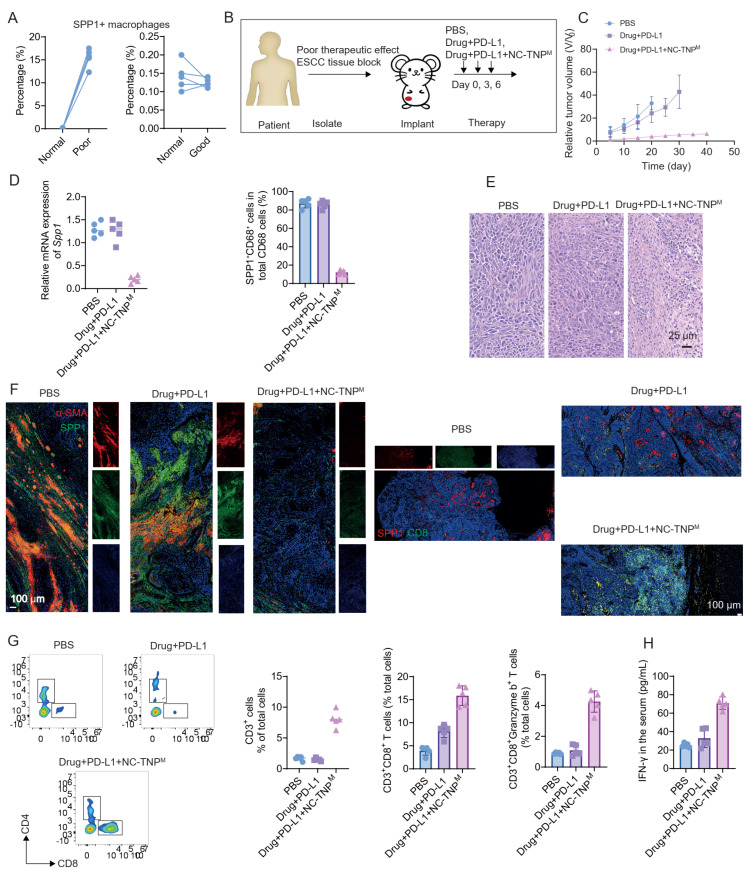
Destruction of the immune barrier formed by SPP1⁺ macrophages and fibroblasts enhances therapeutic efficacy in PDX mice model. (A) Comparison of SPP1⁺ macrophage abundance in paired tumor and adjacent normal tissues from Fn-positive patients with differing responses to neoadjuvant chemo-immunotherapy. Tissue samples were collected from individuals exhibiting either favorable (responders) or poor (non-responders) outcomes. SPP1⁺ macrophage levels were quantified and compared between tumor tissue and corresponding normal tissue. (B) Establishment of a PDX model using tumor tissue from Fn-positive patients with poor response to neoadjuvant chemo-immunotherapy. Tumor specimens were excised, trimmed into 10 × 10 × 10 mm³ fragments, and orthotopically implanted into immunodeficient mice on Day 0 to generate PDX models. Vehicle or treatment regimens were administered via tail-vein injection on Days 0, 3, and 6: (i) PBS control, (ii) Drug (1 mg/kg of cisplatin and 4 mg/kg of paclitaxel were intraperitoneally administered) + anti-PD-L1 (10 mg/kg), and (iii) Drug + anti-PD-L1 + NC-TNP^M^ (5mg/kg for mRNA, and 10 mg/kg for sgRNA). (C) Longitudinal plots of relative tumor volume (normalized to Day 0 = 1.0) for each treatment group. Data are presented as mean ± SD. (D) Analysis of SPP1 expression in PDX tumors and TAMs at day 20. Proportion of SPP1⁺ macrophages (CD68⁺) isolated from tumors, analyzed by RT-qPCR and flow cytometry. (E) H&E staining of resected tumor tissue. (F) Illustrative *ex vivo* murine tumor section images stained to map the spatial arrangement of SPP1-positive macrophages, α-SMA-positive fibroblasts, and CD8-positive T cells within the tumor. (G) After excision, tumor tissues were digested with collagenase to generate a single-cell suspension, which was then analyzed by flow cytometry to determine the proportions of CD3⁺ T cells, CD8⁺ T cells, CD3⁺CD8⁺ T cells, and CD3⁺CD8⁺Granzyme B⁺ T cells (mean ± SD; n = 5). (H) IFN-γ levels in the blood were quantified by ELISA.

The above study elucidates the mechanisms underlying ESCC’s resistance to neoadjuvant chemotherapy combined with immunotherapy. Fn invades the tumor and induces SPP1 overexpression in macrophages. These SPP1-high macrophages interact with fibroblasts, driving their proliferation and together forming an immune barrier. Although this barrier does not alter the overall number of immune cells within the tumor, it remodels their spatial distribution, preventing newly recruited lymphocytes from penetrating the tumor core. As a result, lymphocyte infiltration and expansion are impeded by the immune barrier. Furthermore, we plan to establish PDX mouse models to validate the above-described mechanism and to assess the efficacy of the devised therapeutic strategy. To validate the above mechanism, we performed flow cytometry analysis of SPP1⁺ macrophage content in tumor tissues and adjacent normal tissues from Fn⁺ ESCC patients classified as responders or non-responders to neoadjuvant chemo-immunotherapy. As shown in the Figure 5A, tumor tissues from Fn⁺ non-responders exhibited significantly higher proportions of SPP1⁺ macrophages compared to other groups. This finding directly supports the proposed mechanism. In NSG mice reconstituted with patient-derived CD34⁺ HSCs and bearing ESCC PDXs, we administered two weekly intravenous infusions of *ex vivo*–expanded autologous CD4⁺ and CD8⁺ T cells (2 × 10⁶ cells) once tumors reached ~5 × 5 mm in mice with > 30% human CD45⁺ chimerism. Here designated as day 0, mice received intravenous injections on days 0, 3, and 6 of PBS, Drug (1 mg/kg of cisplatin and 4 mg/kg of paclitaxel were intraperitoneally administered) + anti–PD-L1 (10 mg/kg), and drug + anti–PD-L1 + NC-TNP^M^ (5 mg/kg for mRNA, and 10 mg/kg for sgRNA), respectively (Figure 5B). Tumor relative volume data suggested that NC-TNP^M^ significantly enhanced the antitumor efficacy of Drug + PD-L1 (Figure 5C). At the 20-day time point in the aforementioned model, mice were sacrificed and tumor tissues were harvested. A portion of the tumor tissue was digested with collagenase into a single-cell suspension. SPP1⁺CD68⁺ macrophages were then isolated via magnetic bead sorting, and their abundance was assessed using RT-qPCR and flow cytometry (Figure 5D). Results indicated that the Drug+PD-L1+NC-TNP^M^ group significantly reduced the content of SPP1⁺ macrophages, showing an approximately 8.5-fold decrease compared to the Drug+PD-L1 group. Additionally, hematoxylin and eosin (H&E) staining of tumor tissue sections revealed that the Drug+PD-L1+NC-TNP^M^ group significantly reduced fibroblast content, enhanced immune cell infiltration, and promoted tumor cell apoptosis (Figure 5E). Immunofluorescence suggested that NC-TNP^M^ treatment dramatically depleted SPP1⁺ macrophages (p < 0.001; Figure 5F and Figure S21) and simultaneously diminished CAF density and their barrier integrity. As a result, CD8⁺ T cells in NC-TNP^M^ treated tumors infiltrated deeply and dispersed uniformly throughout the tumor parenchyma. In contrast, tumors receiving only chemotherapy plus anti–PD-L1 retained intact macrophage–fibroblast–CAFs barriers that confined CD8⁺ T cells to the peritumoral stroma, preventing their widespread dispersion. On day 20 post-treatment, tumors were harvested, digested with collagenase into single-cell suspensions, and analyzed by flow cytometry for immune infiltration. Compared with the Drug + PD-L1 group, NC-TNP^M^–treated tumors showed markedly higher levels of intratumoral CD8⁺ T cells, CD3⁺ T cells, CD3⁺CD8⁺ T cells, and CD3⁺CD8⁺Granzyme B⁺ T cells—approximately 6.15-, 2.57-, and 2.70-fold increases for the CD8⁺, CD3⁺, and CD3⁺CD8⁺ populations, respectively (Figure 5G). As shown in Figure 5H, serum immune factor levels were then measured by ELISA, revealing that the NC-TNP^M^ group exhibited a markedly higher IFN concentration.

## Discussion

Recent studies have identified SPP1⁺ macrophages and activated fibroblasts as key drivers of immune-excluded tumor microenvironments across multiple cancers. In colorectal cancer, FAP⁺ fibroblasts and SPP1⁺ macrophages cooperate to establish a desmoplastic niche associated with poor response to PD-L1 blockade 19. Similarly, in hepatocellular carcinoma, SPP1⁺ macrophages and CAFs form the tumor immune barrier (TIB), which limits lymphocyte infiltration and contributes to immunotherapy resistance 18. Here, we identify a comparable stromal program in ESCC and further demonstrate that it is driven by intratumoral Fusobacterium nucleatum (Fn).

Fn promoted macrophage SPP1 expression, leading to CAF activation, extracellular matrix deposition, and formation of a dense stromal barrier. Rather than reducing lymphocyte abundance, this barrier altered their spatial distribution, restricting therapy-induced CD8⁺ T cells to the tumor periphery and limiting cytotoxic activity within the tumor core. These findings identify the Fn–SPP1–CAF axis as a mechanism of immune exclusion underlying resistance to neoadjuvant chemo-immunotherapy. Fn colonization alone, however, was insufficient to induce this phenotype. A subset of Fn-positive tumors remained sensitive to treatment and exhibited low macrophage SPP1 expression, suggesting that additional host-derived signals are required for macrophage reprogramming. Inflammatory cytokines, host genetic background, bacterial strain heterogeneity, and interactions with other microbial species may all contribute and warrant further investigation.

To therapeutically target this pathway, we developed a mannose-modified macrophage-targeted nanoparticle delivering Cas9 mRNA and SPP1-specific sgRNA. Selective SPP1 disruption in tumor-associated macrophages effectively dismantled the stromal barrier, restored intratumoral CD8⁺ T-cell infiltration, and enhanced the efficacy of chemotherapy combined with PD-L1 blockade across multiple humanized PDX models. Several limitations should be acknowledged. Humanized PDX models only partially recapitulate the complexity of human immunity. Although no overt toxicity or systemic inflammatory responses were observed, the long-term safety, biodistribution, off-target editing, and immunogenicity of NC-TNPM require further evaluation before clinical translation 41.

These results also underscore the biological heterogeneity of Fn-positive ESCC. Microbial status alone is unlikely to predict therapeutic response. Instead, SPP1⁺ macrophage abundance together with spatial immune organization may provide mechanistically informed biomarkers for neoadjuvant treatment stratification, a possibility that requires prospective validation. In summary, this study identifies an Fn-driven macrophage–fibroblast circuit that establishes stromal immune exclusion in ESCC. Targeted disruption of macrophage SPP1 restores T-cell penetration and improves chemo-immunotherapy efficacy, supporting macrophage-directed gene editing as a promising strategy for Fn-associated, immune-excluded ESCC and potentially other fibrotic solid tumors.

## Supplementary Material

Supplementary figures and tables.

## Data Availability

The main data supporting the results in this study are available within the paper and its Supplementary Information. All the raw and analyzed data generated during the study are available from the corresponding authors on reasonable request. Source data are provided with this paper.
