# Supplementary material for: Reshaping immune cell distribution with mRNA noncationic lipid nanoparticles for overcoming neoadjuvant chemo-immunotherapy resistance
Source: Theranostics. 2026 Jul 22;16(14):8347–60. doi: 10.7150/thno.136879 (PMC13440642; doi:10.7150/thno.136879)
Supplement: Supplementary file 1 [file thnov16p8347s1.pdf]

Reprogramming immune cell spatial topography with mRNA noncationic lipid nanoparticles for overcoming neoadjuvant chemo-immunotherapy resistance

Yiwen Liu[1,2], Rui Chang[3], Kenan Chen[3], Lin Li[2], Xiaogang An[1], Shegan Gao[4,*], Dingjun Zha[1,*], Hongzhang Deng[1,2,*]

[1]Department of Otolaryngology-Head and Neck Surgery, Xijing Hospital, Air Force Medical University, Xi′ an, Shaanxi 710032, China

[2]Engineering Research Center of Molecular & Neuroimaging Ministry of Education, School of Life Science and Technology, Xidian University, Xi′ an, Shaanxi, 710126, China

[3]Anyang Tumor Hospital, Anyang, Henan, China

[4]College of Clinical Medicine, The First Affiliated Hospital, Henan University of Science and Technology, Luoyang 471003, Henan, China

*Corresponding authors: hzdeng@xidian.edu.cn (H. D.); zhadjun@fmmu.edu.cn (D. Z.); gsg112258@163.com (S. G.)

Keywords: Neoadjuvant chemo-immunotherapy resistance; immune cell spatial topography; mRNA delivery; macrophage; esophageal squamous cell carcinoma

Method

Materials

SM-102 [1-octylnonyl 8-((2-hydroxyethyl)(6-oxo-6-(undecyloxy)hexyl)amino)octanoate], and DSPC [1,2-distearoyl-sn-glycero-3-phosphocholine] were purchased from sigma. DMG-PEG-Mannose was prepared in our lab. mRNA (encoding EGFP, CAS9, and luciferase) were obtained from Apexbio and Trilink bio technologies. D-Luciferinpotassiumsalt was purchased from Apexbio technologies. Wortmannin and chlorpromazine were purchased from Sigma.

Preparation of NC-TNP^{M}

NC-TNP^{M} encapsulating mRNA were formulated by microfluidic mixing using a solution of noncationic thiourea lipids, DMG-PEG-Mannose, and cholesterol (58.5:1.5:40 molar ratio) dissolved in ethanol (6 mg/mL total lipid) and an aqueous mRNA solution (1 mg/mL in ddH_2O) and sgRNA solution (2 mg/mL in ddH_2O). The solutions were rapidly combined at a 1:3 ethanol-to-aqueous volume ratio (flow rates: 0.1 mL/min ethanol stream, 0.3 mL/min aqueous stream) using syringe pumps and a microfluidic chip. The resulting mixture was dialyzed against PBS buffer (pH 7.4) at 4 ºC overnight using a 10 kDa MWCO membrane to remove ethanol and exchange the buffer. Three independent batches of NC-TNP^{M} were prepared on different days using the same microfluidic parameters. Batch characteristics: size (98.2 ± 4.1 nm, 101.5 ± 5.3 nm, 96.8 ± 3.9 nm), PDI (<0.15), encapsulation efficiency (88.4 ± 2.1%, 89.2 ± 1.8%, 87.6 ± 2.5%), and in vitro SPP1 knockdown (>95% for all batches).

Assessment of mRNA Integrity

The integrity of NC-TNP^{M}-encapsulated mRNA is determined through a multi-step analytical process. First, the lipid nanoparticles are disrupted by adding the formulation directly to a denaturing lysis buffer containing guanidinium thiocyanate, followed by thorough vortexing and a brief incubation at room temperature to ensure complete release of the mRNA payload. The RNA is then purified via sequential phenol-chloroform phase separation, precipitated from the aqueous phase with isopropanol in the presence of a glycogen carrier at -20°C, washed with 75% ethanol, and finally resuspended in RNase-free water or TE buffer. For analysis, a small aliquot of the extracted mRNA is denatured in a fluorescent dye-containing sample buffer, heat-treated at 72°C, and analyzed by capillary gel electrophoresis using an instrument such as an Agilent Bioanalyzer or Fragment Analyzer. The resulting electropherogram is evaluated by integrating the area under the curve (AUC) for the intact, full-length mRNA peak; the percentage mRNA integrity is specifically calculated as [AUC of the main peak] / [Total AUC within a defined sizing window] × 100%, providing a

quantitative measure while the electropherogram profile itself offers a qualitative assessment of degradation.

sgRNA sequence

The sgRNA sequences of Spp1 were designed using the online tool platform of Zhang Feng's laboratory (http://chopchop.cbu.uib.no/)

Table S1 the sgRNA sequence

SPP1-Sg1-F	CACCGCCTACAGTCGATGTCCCCAACGG
SPP1-Sg1-R	AAACCCGTTGGGGACATCGACTGTAGG

RAW264.7 cells were transfected with NC-TNP^M^ (Cas9 mRNA + SPP1 sgRNA). After 72 h, genomic DNA was extracted and the SPP1 target region was amplified by PCR (primers: forward 5'-TGGTGGTGACTACCTGCTGT-3', reverse 5'-GGACACACTGCCTCTCCTTC-3'). The PCR product was subjected to Sanger sequencing, and editing efficiency was calculated by TIDE analysis (https://tide.nki.nl/). Knockdown efficiency was about 95% which was confirmed by qRT-PCR.

Gene transfection

GFP-expressing RAW264.7 macrophages were plated in 48-well plates and maintained for 24 hr at 37°C in complete medium (10% FBS). Following PBS washing, cells underwent overnight serum deprivation in FBS-free medium. NC-TNP^{M} nanoparticles co-loaded with mRNA and sgRNA (delivering 1 μg mRNA and 2 μg sgRNA/well) were then applied to cells for 36 h. Transfection efficiency was quantified via flow cytometry and qualitatively assessed by confocal microscopy based on GFP signal intensity.

***In vivo* gene transfection**

All experiments were approved by the Institutional Animal Care & Use Committee (IACUC) of Xidian University.

Subcutaneous injections of mRNA-TNPs (luciferase mRNA payload; 0.1 mg mRNA/kg) of defined rigidity variants were administered to C57BL/6 mice. Luciferase expression was assessed via bioluminescence imaging (IVIS) at 24 h and 48 h post-injection. Mice received an intraperitoneal injection of D-luciferin (150 μL, 10 mg/mL) 10 min prior to each imaging time point.

RT-PCR Analysis

Total RNA was extracted from homogenized tissues/cells using TRIzol reagent, followed by DNase I treatment to eliminate genomic DNA contamination. RNA purity and concentration were determined by spectrophotometry (A260/A280 > 1.8). First-strand cDNA was synthesized from 1 μg total RNA using oligo(dT)18 primers and M-MLV reverse transcriptase at 42°C for 60 min, with a final enzyme inactivation step at 70°C for 15 min. PCR amplification was performed in 25 μL reactions containing 2 μL

cDNA template, gene-specific primers (0.2 μM each), dNTPs (200 μM), and Taq DNA polymerase (1 U). Thermal cycling conditions consisted of: initial denaturation at 95°C for 5 min; 35 cycles of 95°C for 30 sec, 72°C for 45 sec; and final extension at 72°C for 10 min. Amplified products were electrophoresed on 1.5% agarose gels stained with ethidium bromide, with β-actin serving as the internal control.

ELISA for Cytokine Detection

Cell culture supernatants or tissue homogenates (prepared in PBS containing protease inhibitors and centrifuged at 12,000 × g for 15 min at 4°C) were analyzed using sandwich ELISA. High-binding 96-well plates were coated overnight at 4°C with capture antibody (1–5 μg/mL in carbonate-bicarbonate buffer, pH 9.6), then blocked with 5% BSA/PBS for 2 h at room temperature. Samples and recombinant cytokine standards (serially diluted in assay diluent) were incubated for 2 h at RT. After washing (0.05% Tween-20/PBS), plates were incubated with biotinylated detection antibody (1–2 μg/mL in 1% BSA/PBS, 1 h, RT), followed by streptavidin-HRP (30 min, RT). Color development used TMB substrate (15 min, dark), stopped with 2N H_2SO_4. Absorbance was measured at 450 nm (reference 570 nm) using a microplate reader, with cytokine concentrations interpolated from the standard curve. All samples were assayed in technical duplicates with negative controls (blank, unstimulated samples).

FACS sorting

Single-cell suspensions from digested tumor tissues were stained with fluorochrome-conjugated antibodies (all from BioLegend) for 30 min at 4 °C: for macrophages, CD45-FITC, CD11b-PE, and F4/80-APC (or CD68-APC for human samples); for tumor cells, EpCAM-PE/Cy7 and CD45-FITC (negative); for fibroblasts, α-SMA-Alexa Fluor 488 with CD45-FITC and EpCAM-PE/Cy7 negativity; for T cells, CD45-FITC, CD3-PerCP/Cy5.5, and CD8-PE. Sorting was performed on a BD FACSAria III with a 100 μm nozzle using purity mode (target >98%). The gating hierarchy was applied sequentially: first, debris was excluded by FSC-A vs. SSC-A; second, single cells were selected by FSC-A vs. FSC-H; third, live cells were gated using Zombie Aqua™ viability dye (negative population); fourth, $CD45^+$ leukocytes were gated for macrophage and T-cell sorting, while for tumor cells and fibroblasts the $CD45^-$ gate was used; fifth, lineage-specific gates were drawn: macrophages as $CD45^+CD11b^+F4/80^+$ (or $CD68^+$ for human), tumor cells as $EpCAM^+CD45^-$, fibroblasts as α-$SMA^+CD45^-EpCAM^-$, and $CD8^+$ T cells as $CD45^+CD3^+CD8^+$. Sorted cells were collected directly into PBS containing 0.1% BSA for downstream DNA extraction.

Immediately after FACS sorting, macrophages were pelleted (10,000×g, 5 min) and DNA was extracted using the Qiagen DNeasy Blood & Tissue Kit according to the manufacturer's protocol, including an optional RNase A step; to release intracellular

bacterial DNA, an additional 15 min incubation with 0.1% Triton X-100 was performed before lysis. Multiple contamination controls were included: a no-sort control (cells processed through the entire staining and sorting workflow but not sorted) to assess carryover, DNase I treatment (10 U/μL, 37 °C, 30 min) before DNA extraction to remove any residual extracellular bacterial DNA, a negative extraction control (PBS processed identically to cell samples), and a no-template control in qPCR. Quantitative PCR for Fusobacterium nucleatum used primers targeting the 16S rRNA gene (forward: 5'-CCTTGGCCATATACTCAGGT-3', reverse: 5'-GCAGTTACTCCACCTTCCTTC-3') and a FAM-labelled probe (5'-TGGACGCAAGTCTG-3'). A standard curve was generated with serial dilutions of purified Fn genomic DNA (10^0 to 10^5 copies/μL), and results were normalized to host β-actin DNA copies, expressed as Fn copies/μg DNA.

Immunofluorescence Staining of Tumor Tissues

Formalin-fixed paraffin-embedded (FFPE) tumor sections (4–5 μm) were dewaxed in xylene and rehydrated through graded ethanol. Antigen retrieval was performed by pressure-cooking in citrate buffer (pH 6.0) for 20 min. After cooling and PBS washes, sections were permeabilized with 0.1% Triton X-100 (10 min), then blocked with 5% normal serum from the secondary antibody host species (1 h, RT). Primary antibodies diluted in blocking buffer were incubated overnight at 4°C in a humid chamber. Following PBS-Tween 20 washes, fluorophore-conjugated secondary antibodies (e.g., Alexa Fluor 488/594, 1:500) and DAPI (1 μg/mL) were applied for 1 h at RT (light-protected). Sections were coverslipped using antifade mounting medium and imaged by confocal microscopy (Leica SP8), with Z-stacks acquired using sequential scanning to prevent fluorophore bleed-through. Appropriate controls (isotype, no primary antibody, fluorescence-minus-one) were included for validation. Image analysis was performed using Imaris 9.7 with background subtraction.

The flow cytometry-based detection of immune cell infiltration

Following immediate collection into cold PBS/2% FBS, tumor tissue is mechanically minced with scalpels, then enzymatically digested (1 mg/mL Collagenase IV, 0.1 mg/mL DNase I in RPMI) for 30-45 minutes at 37°C with agitation. The resulting suspension is filtered (70 μm), pelleted (300g, 5 min), and subjected to ACK lysis (2-5 min, RT) to remove erythrocytes. Cells are washed, counted, and viability assessed (Trypan Blue). Approximately 1-5x10^6 cells are Fc-blocked (human FcR blocking reagent, 10 min, 4°C), then surface-stained with titrated antibody cocktails in FACS buffer (30 min, 4°C, dark). For intracellular targets, cells are fixed/permeabilized before intracellular staining. A viability dye is incorporated pre-staining. Cells are washed twice, resuspended in FACS buffer with DAPI (1 μg/mL), filtered (35 μm), and

acquired immediately on a flow cytometer calibrated daily using compensation beads.

Flow cytometry protocol for quantifying immune cell subsets in peripheral blood

Peripheral blood collected in EDTA/heparin tubes is processed within 24 hours. Whole blood (100–200 µL) is aliquoted, treated with red blood cell lysis buffer, washed, and resuspended in FACS buffer (PBS/2% FBS). Alternatively, PBMCs are isolated via density gradient centrifugation (Ficoll-Paque™, 400g, 30 min RT), washed twice, and counted. Cells (1×10^6) are Fc-blocked (10 min, 4°C), then surface-stained with pre-titrated antibody cocktails targeting pan-leukocyte in the dark (30 min, 4°C). Viability dye is added pre-staining. For intracellular targets cells are fixed/permeabilized prior to staining. After final washes, cells are resuspended in FACS buffer with DAPI (1 µg/mL), filtered (35 µm), and acquired immediately on a flow cytometer calibrated using compensation beads.

Establishing a PDX mouse model

Following surgical resection, fresh ESCC tumor tissue is immediately placed in cold preservation medium (RPMI-1640 with 10% FBS/1% penicillin-streptomycin) and transported on ice to the lab within 1 hour. Under sterile conditions, necrotic areas are dissected away, and viable tumor tissue is minced into 2-3 mm^3 fragments in PBS. Fragments are either cryopreserved in 90% FBS/10% DMSO or directly mixed 1:1 with growth factor-reduced Matrigel®. Six-to-eight-week-old mice are anesthetized, and 3-4 fragments are surgically implanted subcutaneously into right flank using a trocar. Serum from the same patient is administered intraperitoneally (100 µL/mouse) during engraftment. Mice are monitored twice weekly for tumor growth (caliper measurements), with successful engraftment confirmed at >100 mm^3 volume.

Mice were randomly assigned to treatment groups using a computer generated random number sequence (Random.org). Allocation was performed by an investigator not involved in the experiments. Blinding: All outcome assessments (tumor volume measurements, flow cytometry, immunofluorescence quantification, and histopathological evaluation) were performed by investigators blinded to group allocation. Blinding codes were broken only after all data were collected and analyzed. NSG mice were used for PDX engraftment. To reconstitute a functional human immune system, mice were irradiated (150 cGy) and intravenously injected with 1×10^5 $CD34^+$ hematopoietic stem cells isolated from the same ESCC patient's peripheral blood (Miltenyi CD34 MicroBead Kit). Human $CD45^+$ chimerism in peripheral blood was monitored every 4 weeks by flow cytometry. Only mice with >30% human $CD45^+$ cells (of which 8-12% were $CD3^+CD8^+$ T cells) were used for PDX implantation. Tumor fragments ($2 \times 2 \times 2$ mm^3) from the same patient were subcutaneously engrafted into humanized NSG mice. This model supports functional human T-cell responses and has been validated for anti-PD-L1 studies.

In situ induction of esophageal squamous cell carcinoma (ESCC) with Fusobacterium nucleatum (Fn) infection in mice

Six-week-old C57BL/6 mice receive 4-nitroquinoline 1-oxide (4NQO) in drinking water (100 μg/mL, pH 4.5) ad libitum for 16 weeks to induce esophageal carcinogenesis. At week 8, broad-spectrum antibiotics (ampicillin 1 mg/mL + metronidazole 0.5 mg/mL) are administered in drinking water for 72 hours to suppress commensal flora. Once visible esophageal tumors or advanced dysplastic lesions are present (typically ~20 weeks), mice are administered Fusobacterium nucleatum via repeated intraluminal injection (e.g., 10^8 CFU in PBS, 2–3 doses per week for several weeks) or gavage depending on study design. This Fn infection is confirmed by qPCR in tumor tissue, and tumor progression is subsequently monitored by endoscopy or histology.

Establishing a murine-derived esophageal squamous cell carcinoma (ESCC) subcutaneous tumor model with Fusobacterium nucleatum (Fn)

Syngeneic murine ESCC cells are cultured in DMEM/10% FBS. Cells harvested at 80% confluency (Trypan Blue viability >95%) are suspended in PBS:Matrigel® (1:1, 4°C) and injected subcutaneously (1×10^6 cells/100 μL) into the right flank of 6-week-old immunocompetent C57BL/6 mice. When tumors reach 50 mm^3 (volume = ½ × length × $width^2$), mice receive intratumoral injections of 5×10^7 CFU anaerobic-cultured Fn suspended in reduced thioglycolate broth every 48 hours for 1 week. Control groups receive vehicle or heat-killed Fn. Tumor growth is monitored 3×/week via caliper measurements.

Statistical analyses

Data are reported as mean ± standard de*via*tion (SD). Statistical analyses were conducted using GraphPad Prism 9 software. Differences between groups were assessed using two-tailed Student's t-test or one-way analysis of variance (ANOVA). A p-value greater than 0.05 indicates no significant difference. Statistical significance levels are denoted as follows: * $p < 0.05$, ** $p < 0.01$, *** $p < 0.001$, **** $p < 0.0001$.

Sample size calculation: Based on our preliminary data, we used a two-sided significance level $\alpha = 0.05$ and power $(1-\beta) = 0.80$ to calculate the minimum number of mice per group. For tumor growth inhibition assays, we estimated an effect size (Cohen’s d) of 1.5 from pilot experiments (difference in mean tumor volume between treated and control groups relative to pooled SD), yielding n = 5–6 per group.

Group setup logic: Subcutaneous ESCC model (Figure 4): Mice were randomized after Fn exposure using a computer-generated random number sequence. Groups included: (i) PBS control, (ii) Chemotherapy + anti-PD-L1, (iii) NC-TNP^M alone, (iv) Combination (Chemo+anti-PD-L1+NC-TNP^M). Randomization was stratified by initial tumor volume to ensure comparable baselines.

PDX model (Figure 5): PDX-bearing NSG mice with human CD34+ HSC

reconstitution were randomized similarly, with groups as described. Blinded outcome assessment was performed for tumor volume measurements and flow cytometry.

Clinical samples

This study selected 230 paraffin-embedded specimens of ESCC tissue from the Department of Pathology, Anyang Tumor Hospital as the research subjects. After routine fixation, dehydration, clearing, and embedding, the ESCC tissues were sectioned (3 μm thick). This study was conducted with patient informed consent and approved by the Ethics Committee of Anyang Tumor Hospital. The inclusion and exclusion criteria are shown in Figure S1. A total of 225 ESCC tissue specimens were finally included, and detailed clinical baseline data of the patients are presented in Supporting EXCEL. The enrolled patients included 20 cases with clinical stage I, 83 cases with stage II, and 122 cases with stage III. The Kaplan-Meier method was used to plot patient survival curves, and the overall survival time between Fn infection-positive and negative patients was compared using the Log-rank test. The clinical follow-up period in this study was set at 5 years. Survival time was defined as the time from confirmed diagnosis at admission to the last follow-up or death. Censored data referred to patients who were still alive at the end of the 60-month follow-up period, while uncensored data referred to patients who died from ESCC. The detail information was supplied as shown in Supporting information EXCEL.

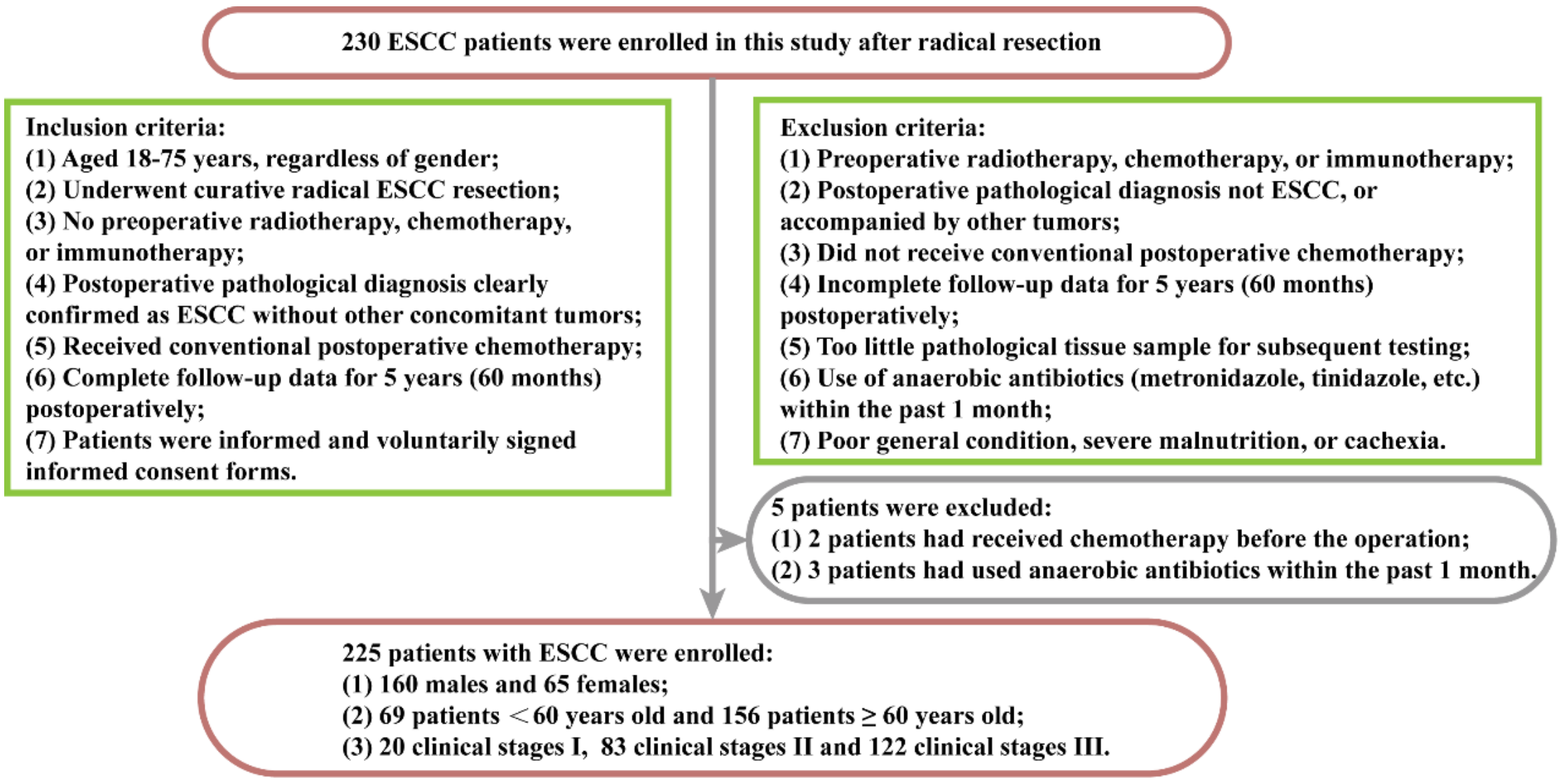

Figure S1 Enrollment criteria of ESCC patients in this study.

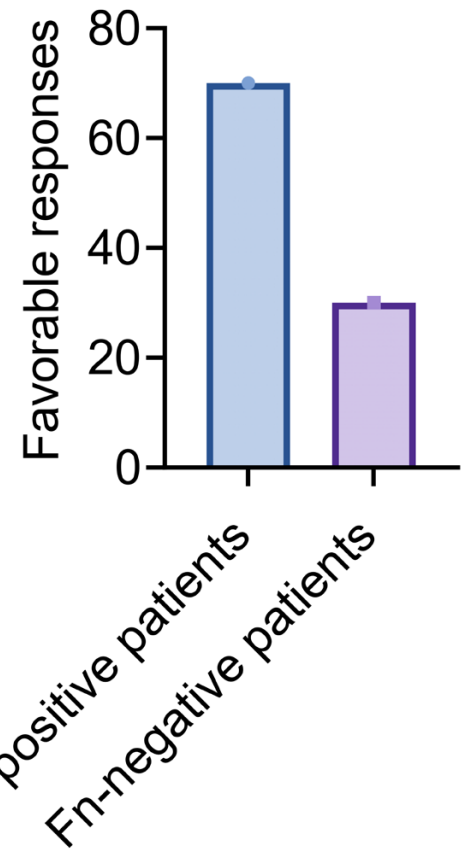

Figure S2 Favorable responses of Fn-negative responders and non-responders patients.

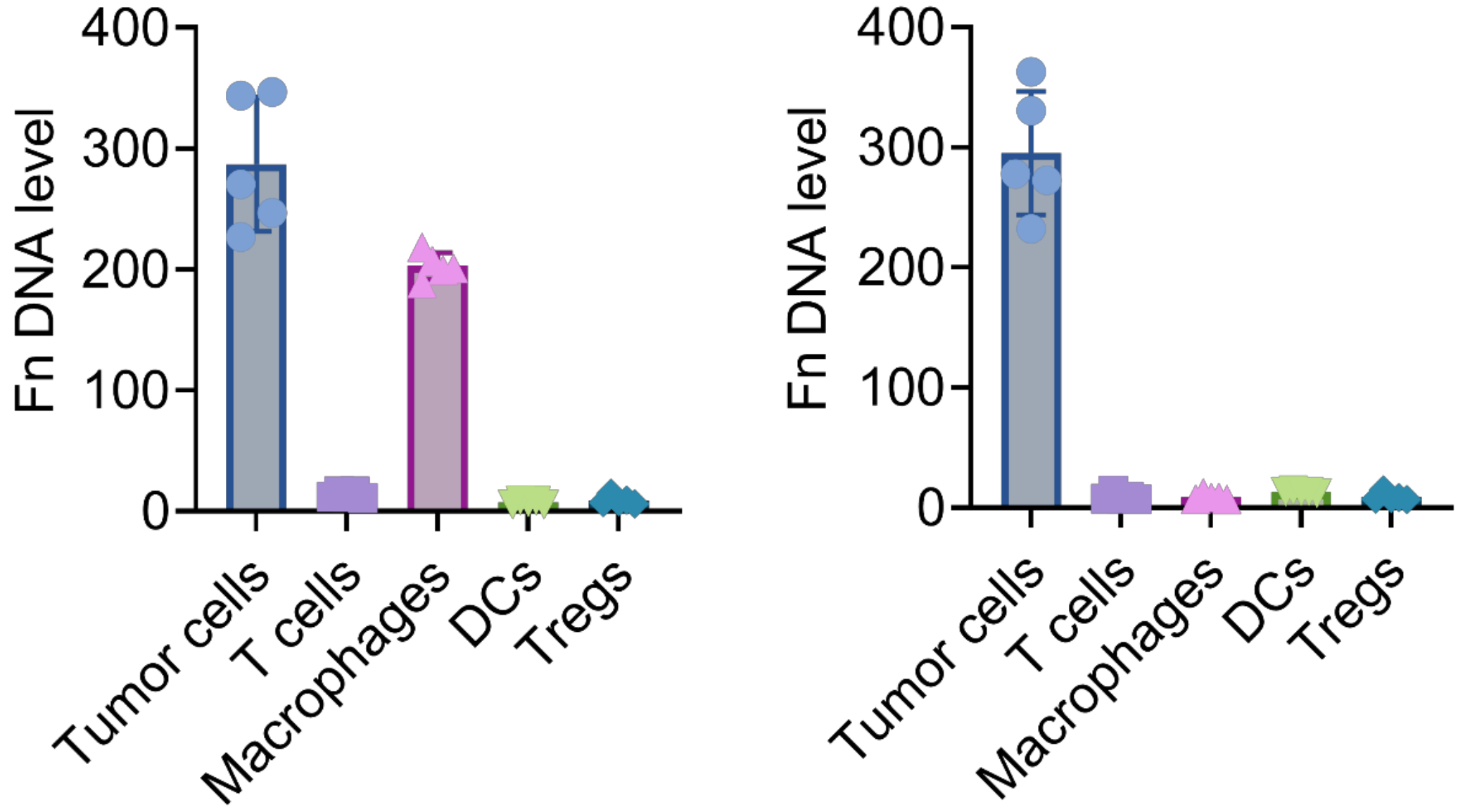

Figure S3 Fn DNA copies were quantified by qPCR in FACS-sorted cells (purity >97%).

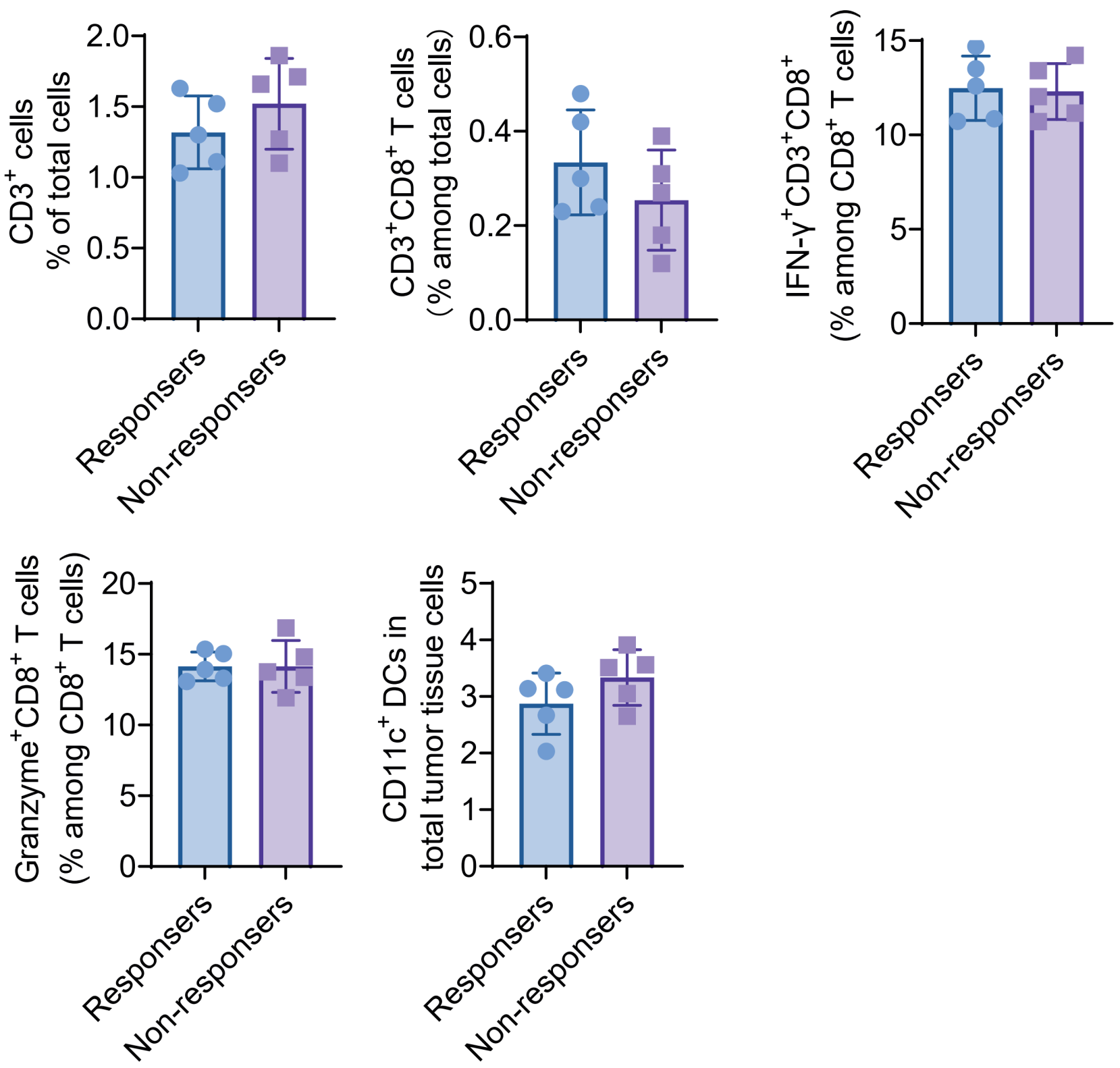

Figure S4 Flow cytometry analysis of immune cell populations in single-cell suspensions from resected post-treatment tumors from untreated groups.

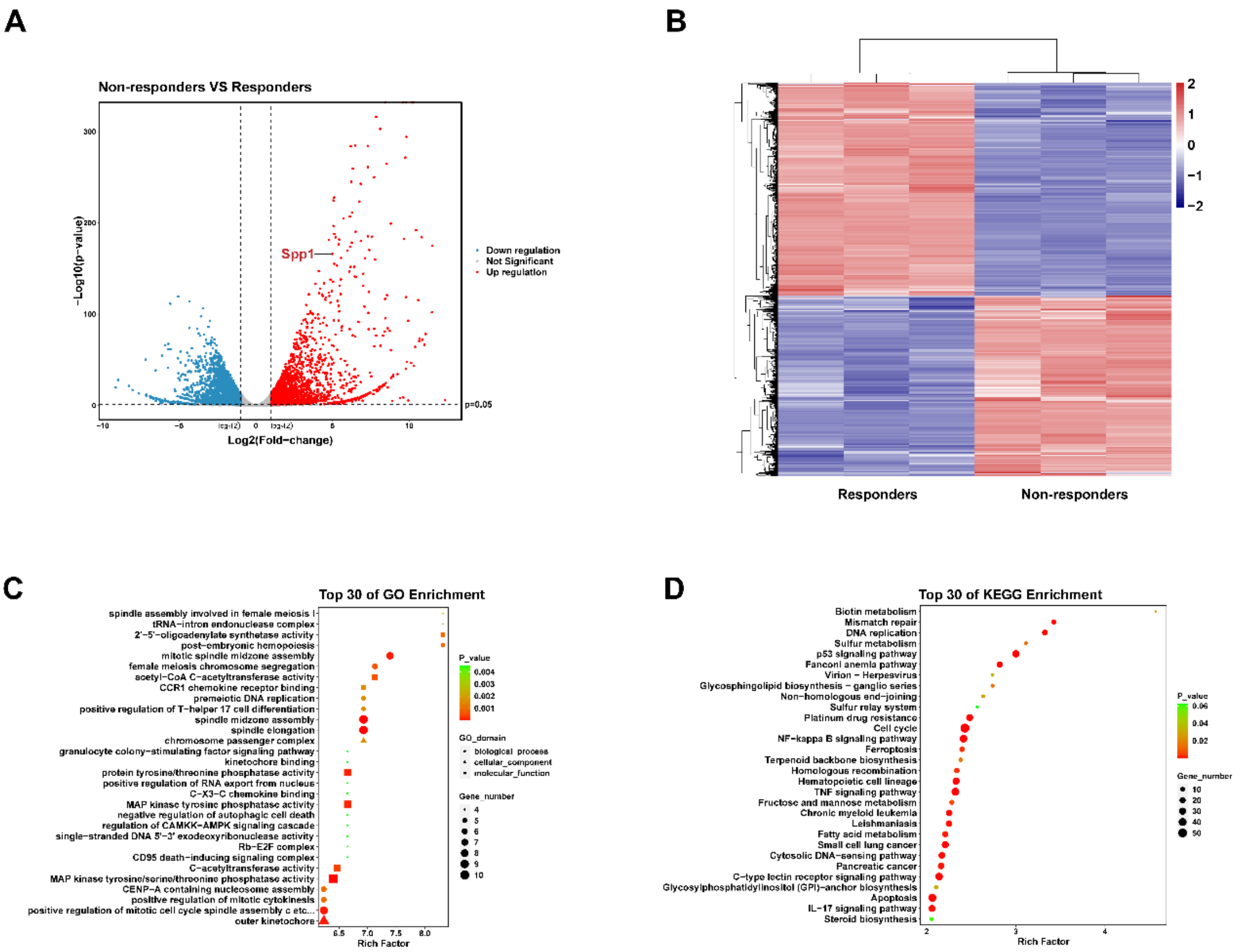

Figure S5 Transcriptome sequencing analysis results. (A) Volcano plot of differentially expressed genes; (B) Heatmap of candidate differentially expressed genes; The screening criteria for differentially expressed genes in this project were: p-value < 0.05 and fold change ≥ 2 (with FPKM > 10 in at least one group); (C) GO enrichment results of differentially expressed genes; (D) KEGG enrichment results of differentially expressed genes.

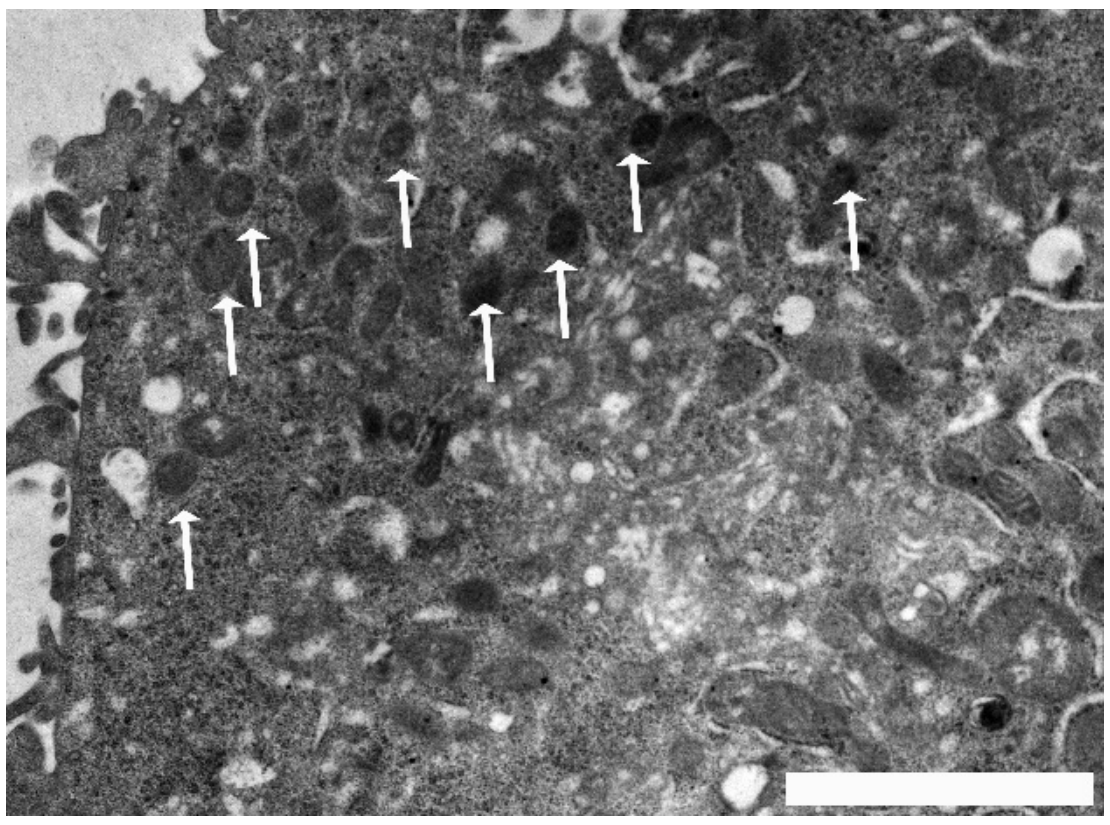

Figure S6 Transmission electron microscopy of macrophages following 48h co-culture with Fn, showing bacterial internalization. Scale bar 2 μm.

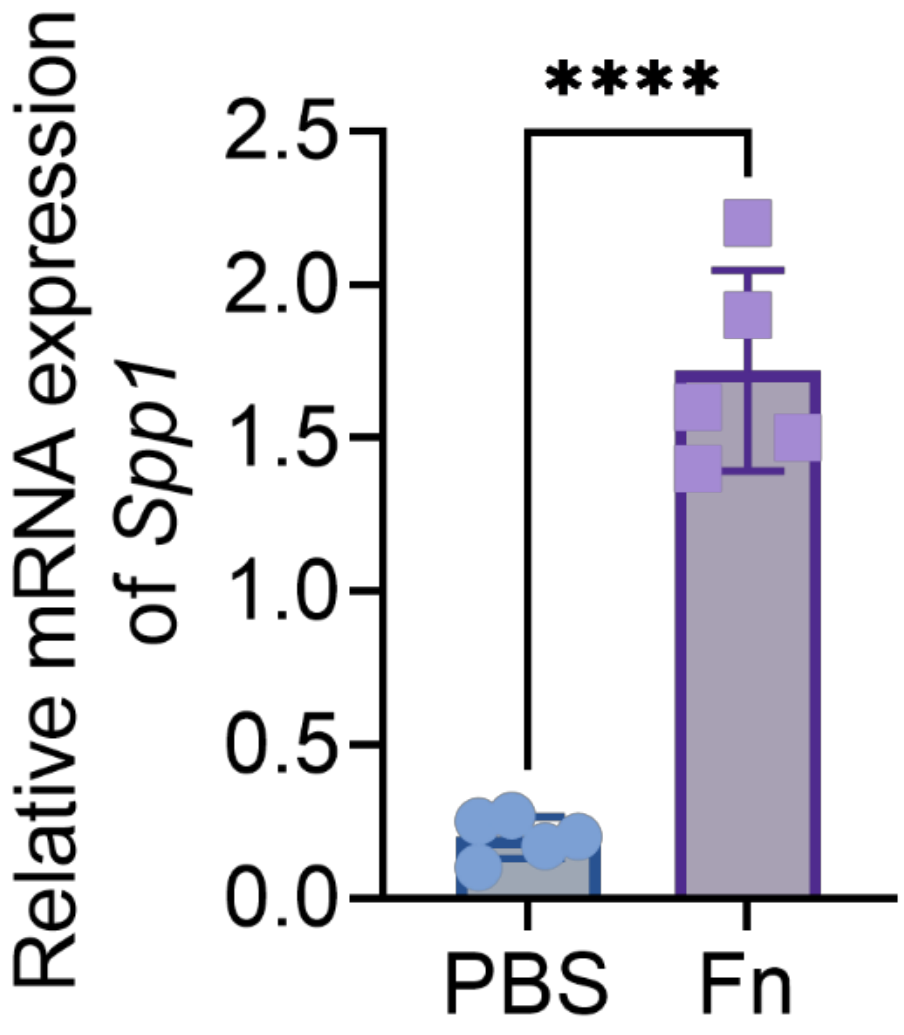

Figure S7 RT-qPCR analysis of SPP1 mRNA in macrophages after 48h co-culture with Fn.

Table S1 Nanoparticle characterization

Encapsulation efficiency	Drug loading capacity	Serum stability
98%	1/20	90 days

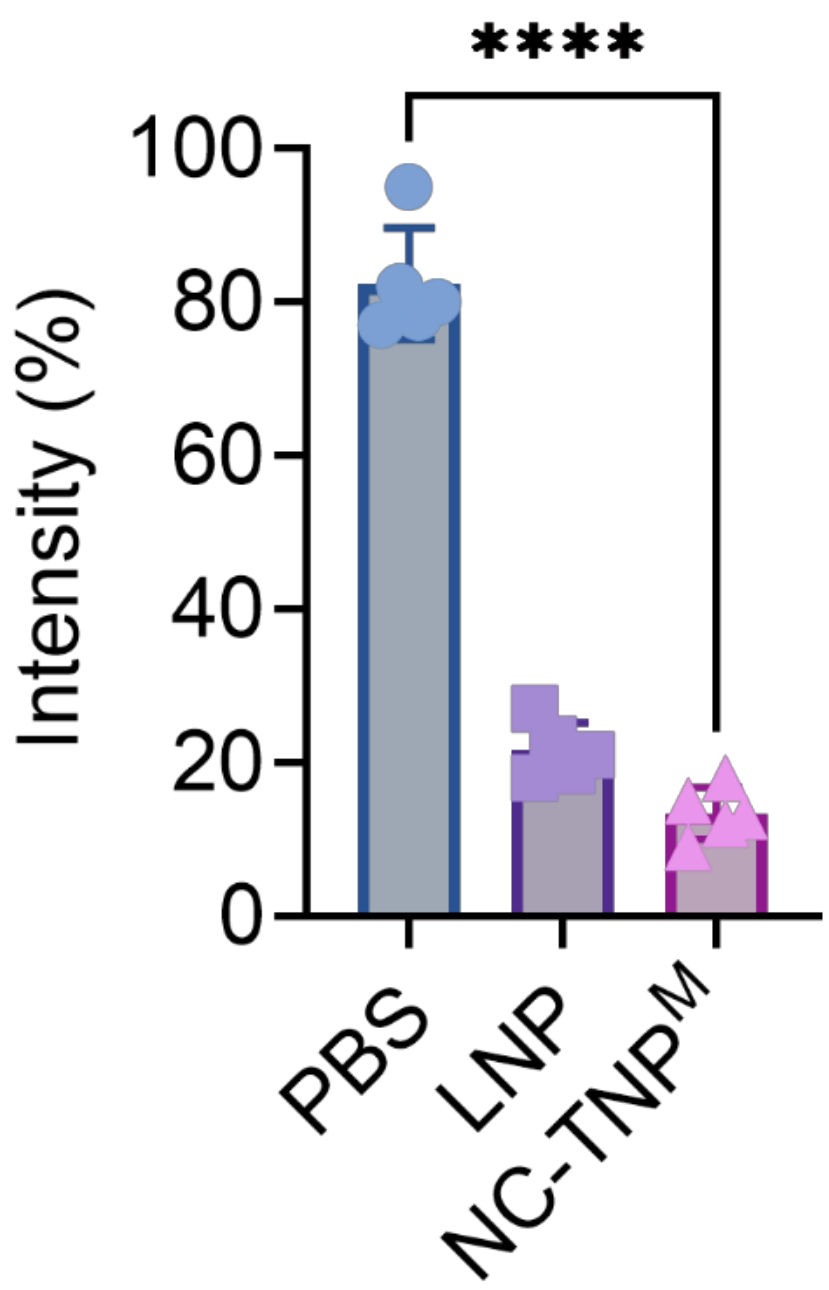

Figure S8 Quantitative fluorescence measurements.

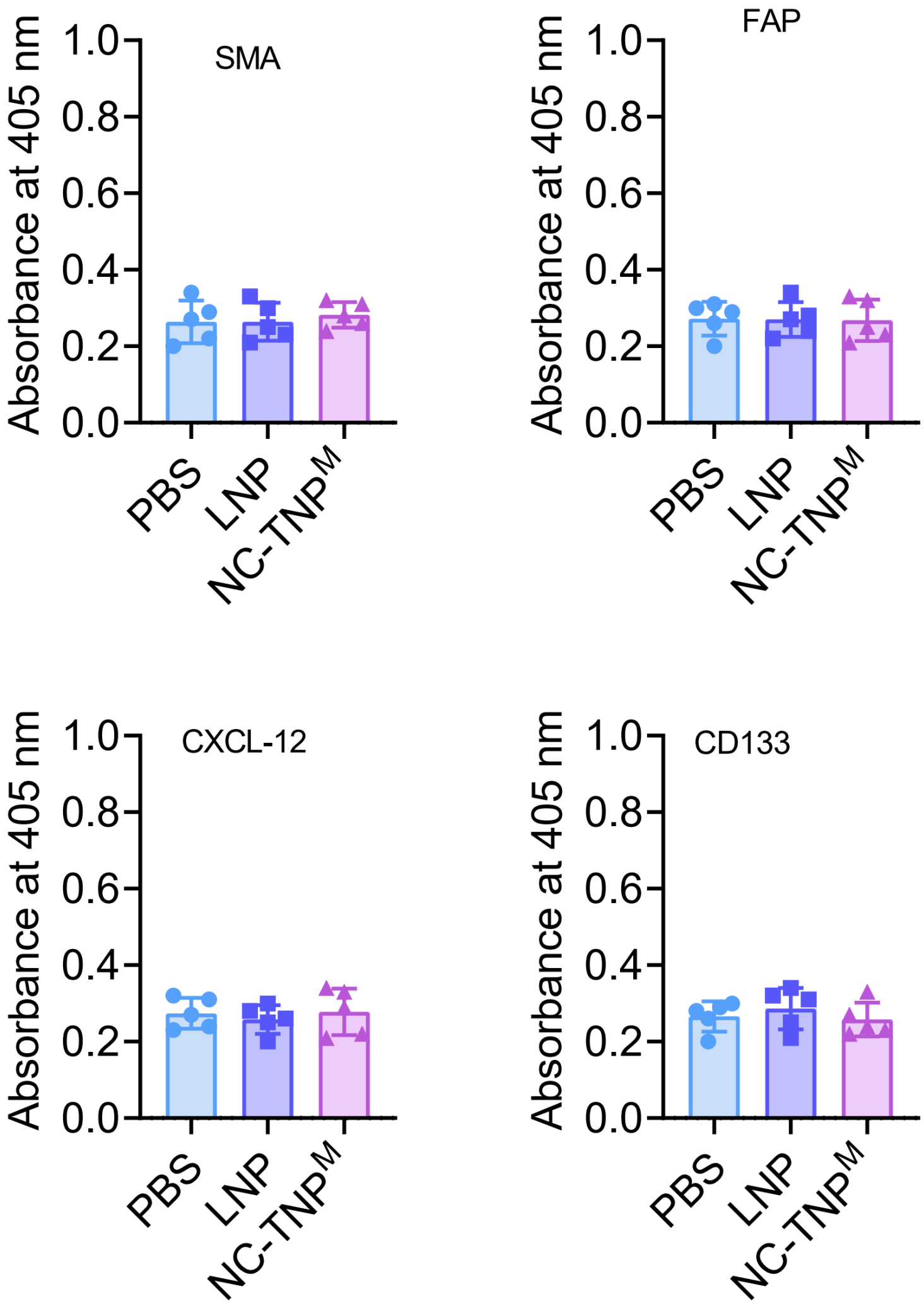

Figure S9 CAF-associated biomarkers (SMA, FAP, CXCL12, and CD133) were quantified by ELISA, knocked down SPP1 in Fn-infected macrophages and co-cultured with cancer-associated fibroblasts, results presented as optical density at 450 nm.

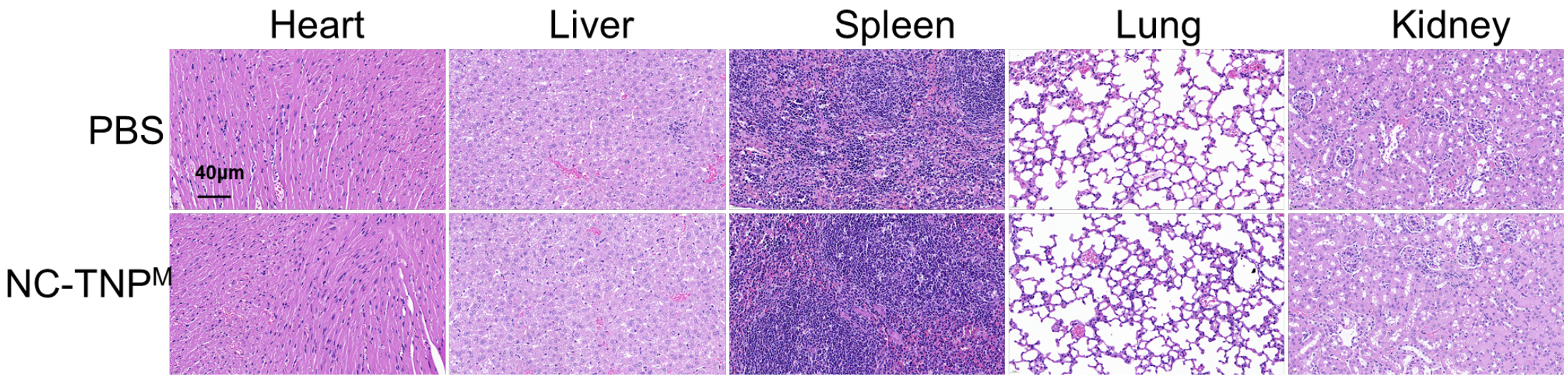

Figure S10 H&E staining of major organs

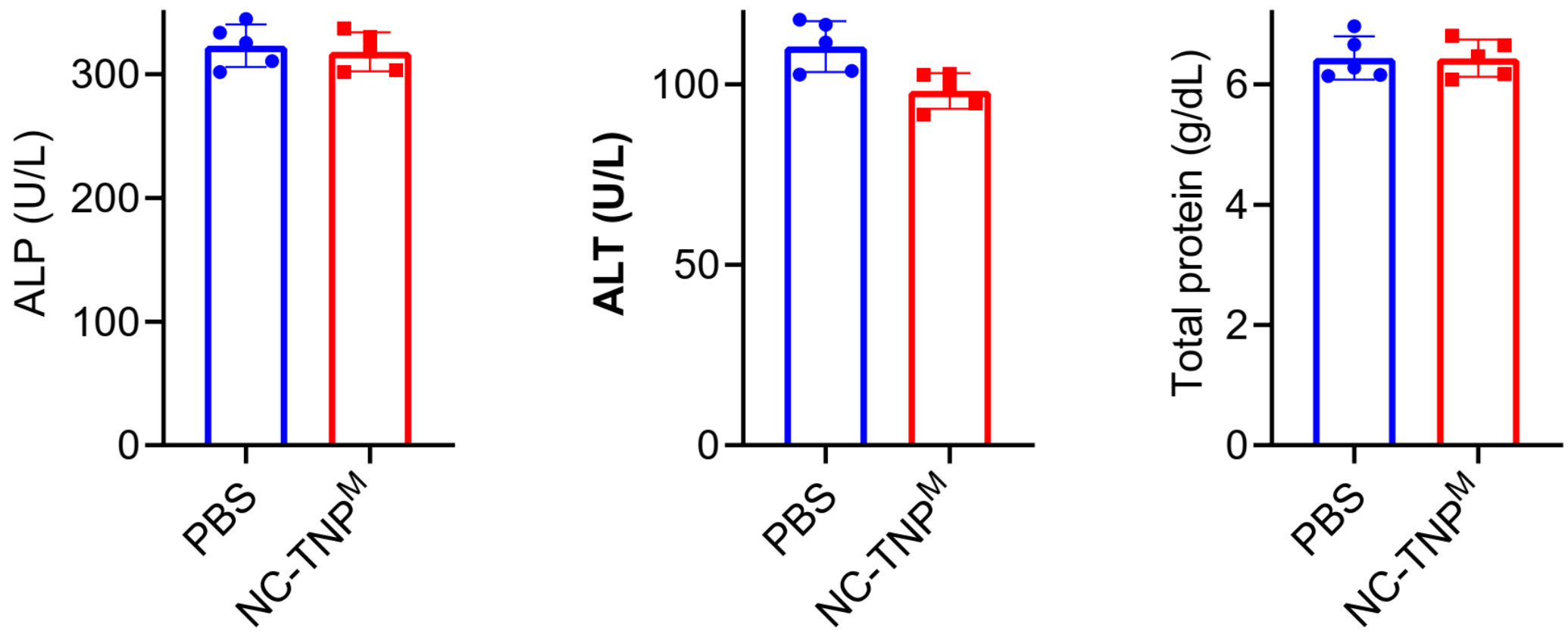

Figure S11 The elevation of liver ALP and ALT activity and total protein.

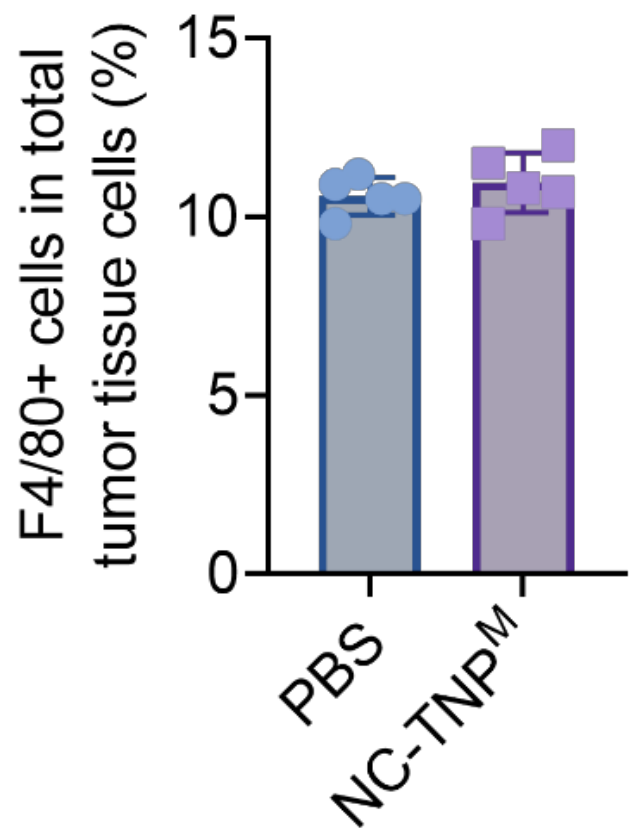

Figure S12 Quantitative flow cytometry was performed to determine total F4/80^{+} macrophage counts in day 0.

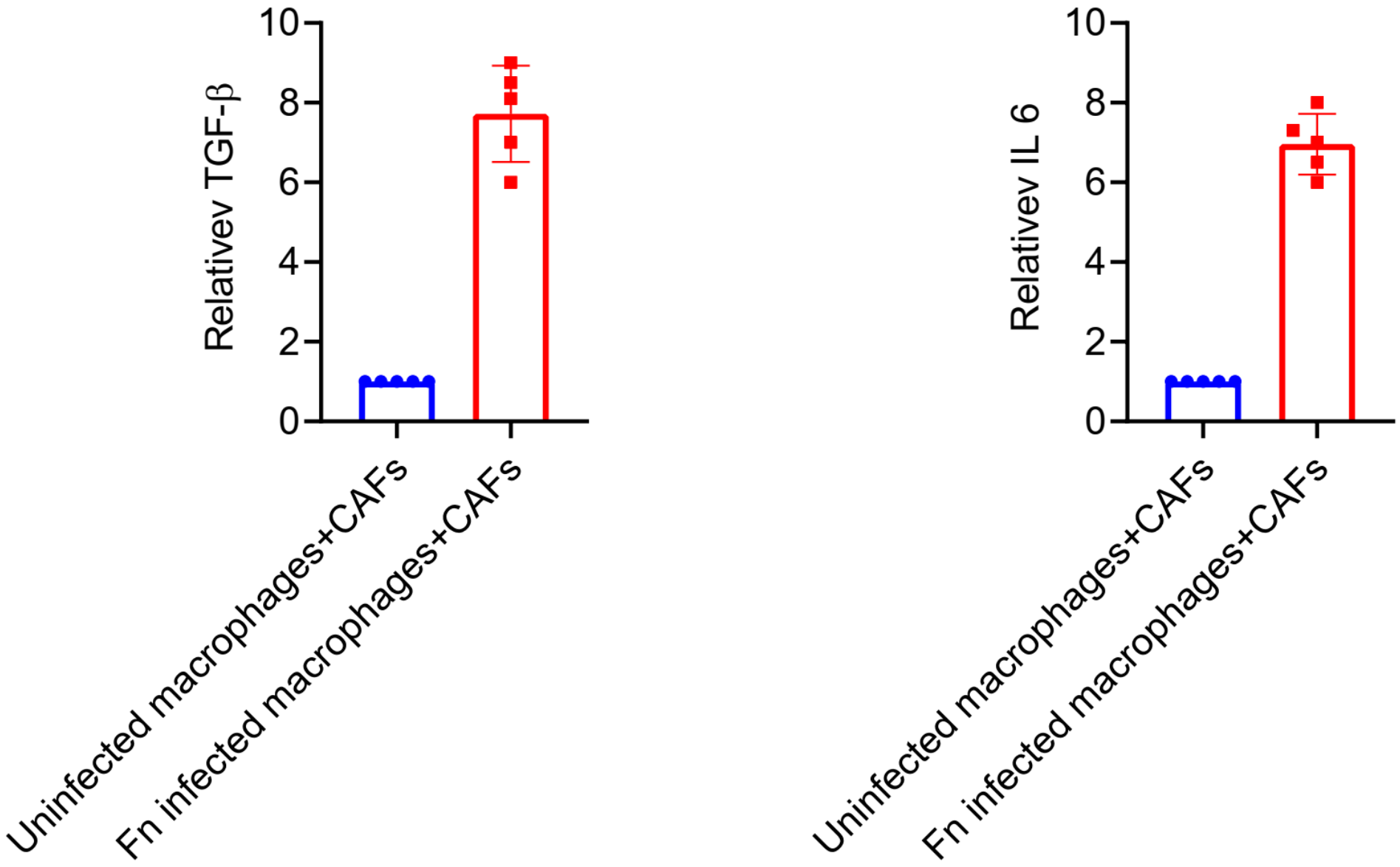

Figure S13 Quantification of cytokines and soluble factors in co-culture supernatants by ELISA, showing increased secretion of TGF-β and IL-6 compared to control groups.

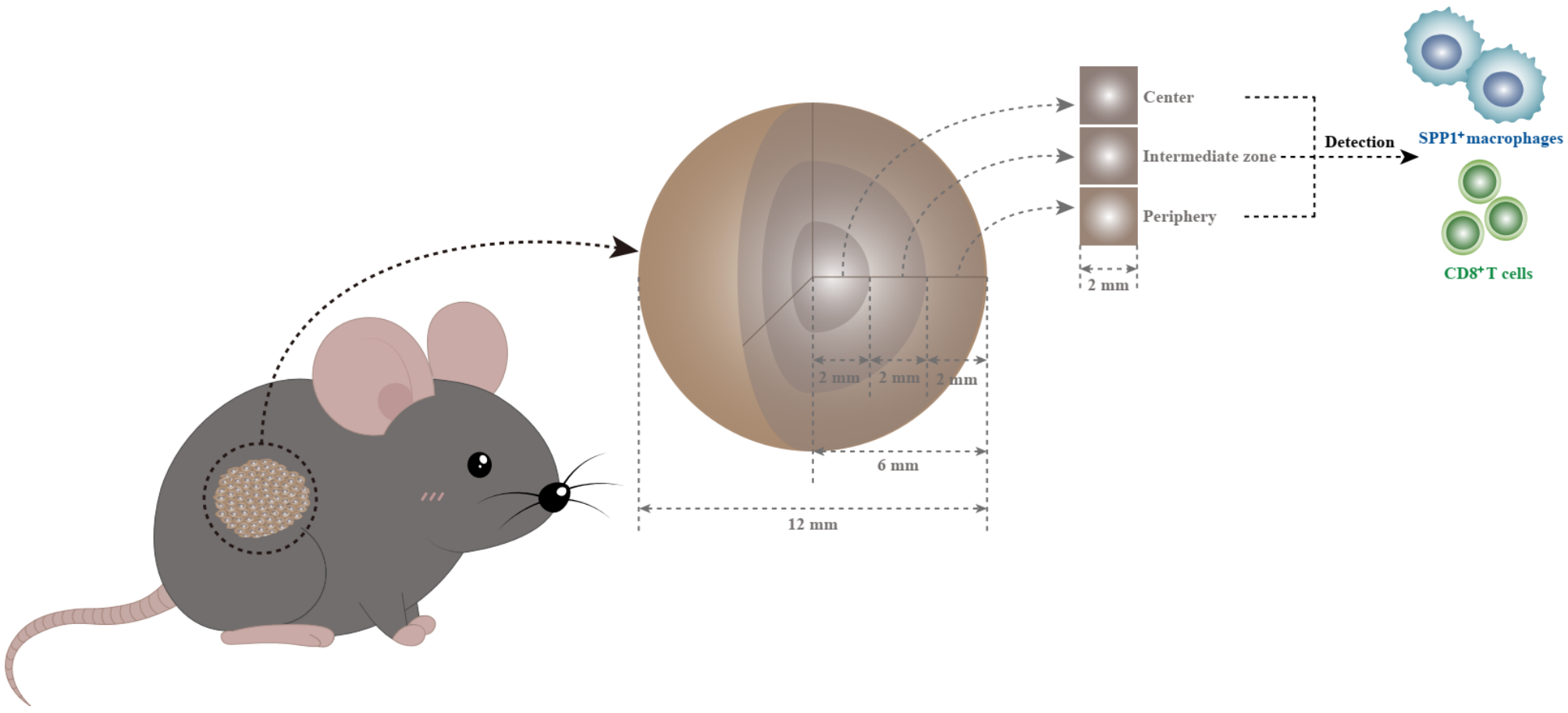

Figure S14 Schematic diagram of the radial sampling scheme for spatial immune profiling of tumors. When tumors reached approximately 1.2 cm in diameter, they were harvested. Starting from the geometric center of the tumor, serial tissue blocks (2 mm × 2 mm each) were collected along outward radial directions (indicated by arrows). This design enabled systematic comparison of immune response profiles across different spatial regions, from the tumor core to the periphery.

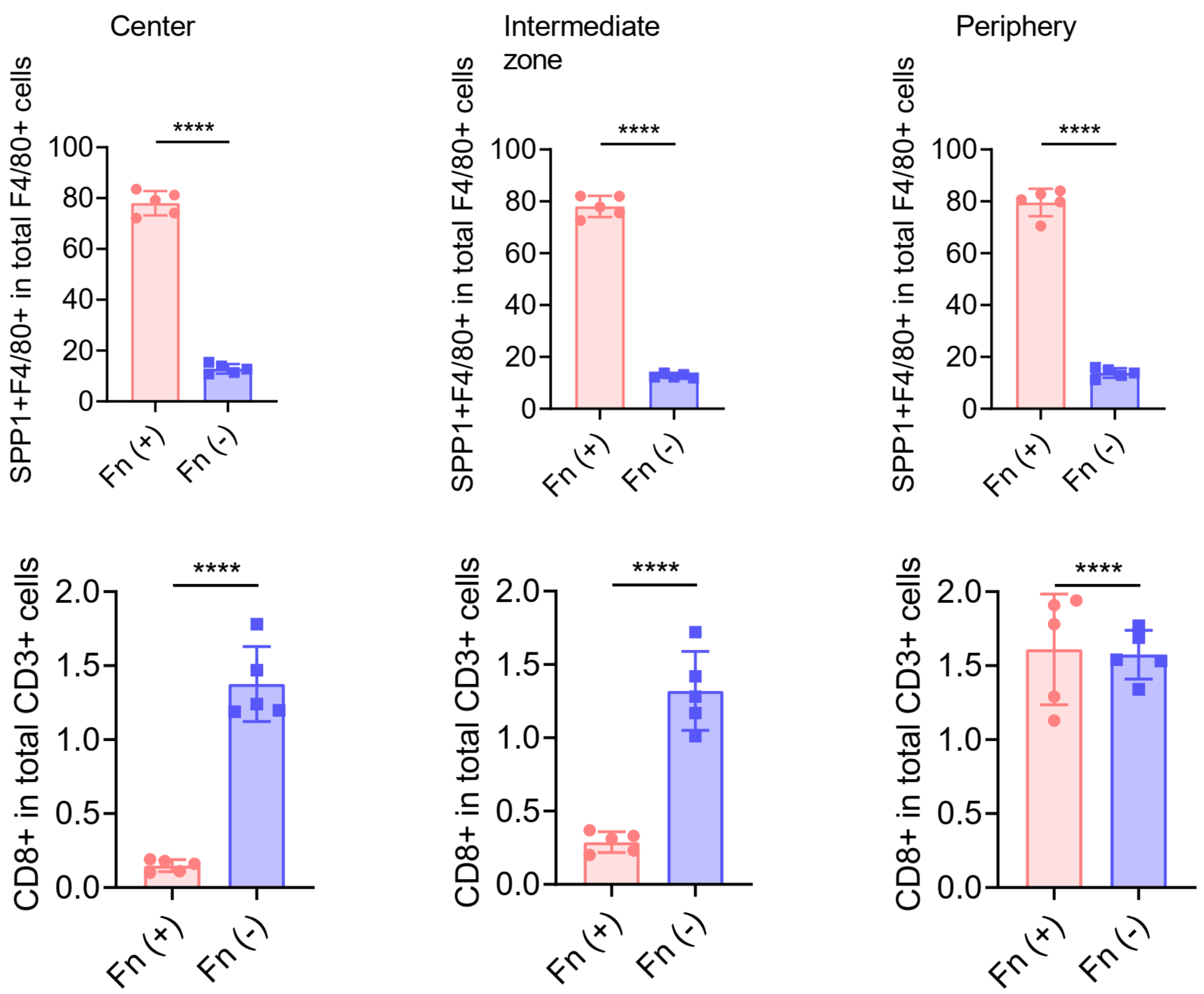

Figure S15 SPP1^{+} macrophages were significantly more abundant in Fn-positive tumors than in Fn-negative tumors. Immune cell distribution was spatially uniform in Fn-negative tissues, but markedly reduced in the central region of Fn-positive tumors, indicating spatial heterogeneity.

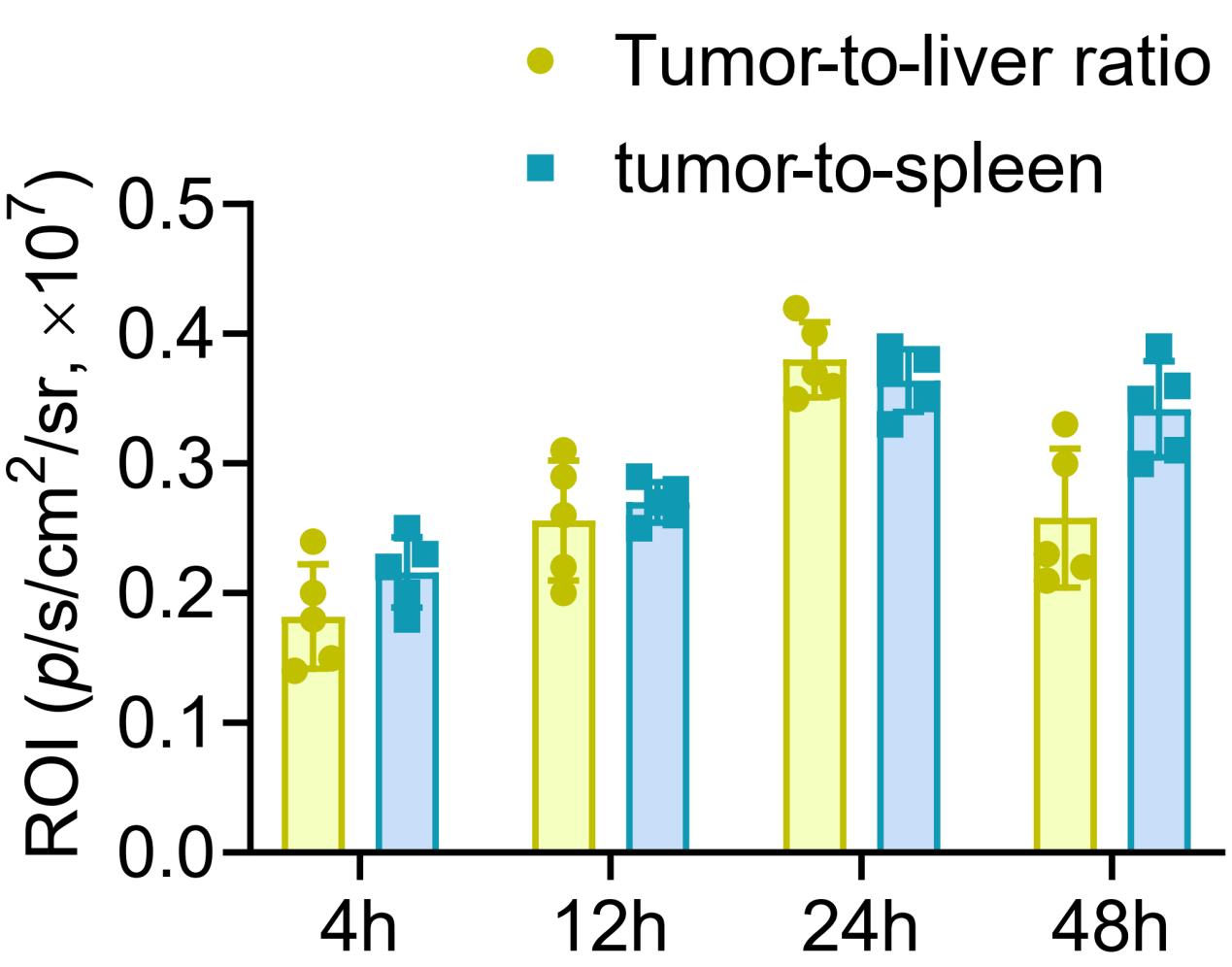

Figure S16 Cy5-labeled NC-TNPM showed time-dependent the tumor-to-spleen and tumor-to-liver ratio.

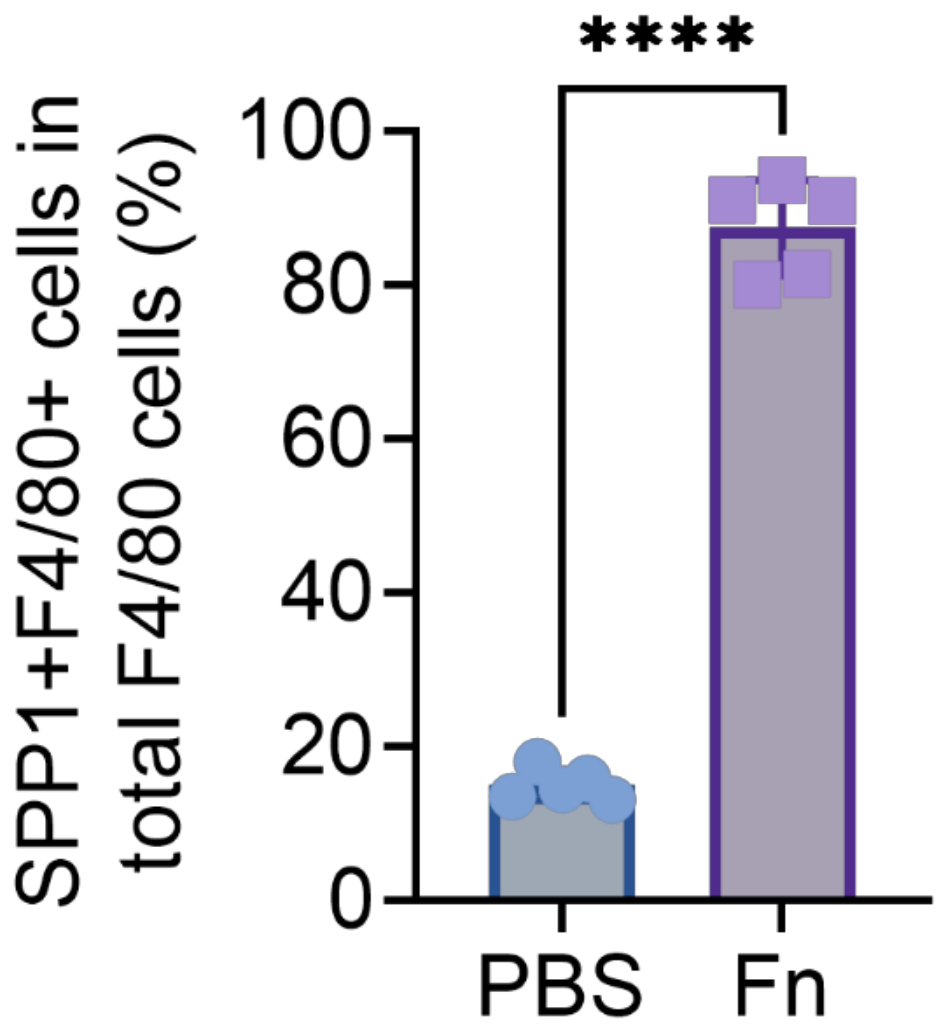

Figure S17 C57BL/6 mice bearing subcutaneous mouse-derived ESCC tumors received PBS and Fn (1×10^8 CFU, multipoint intratumoral, twice weekly × 2 weeks). Quantitative flow cytometry was performed to determine SPP1+F4/80$^+$ macrophage counts.

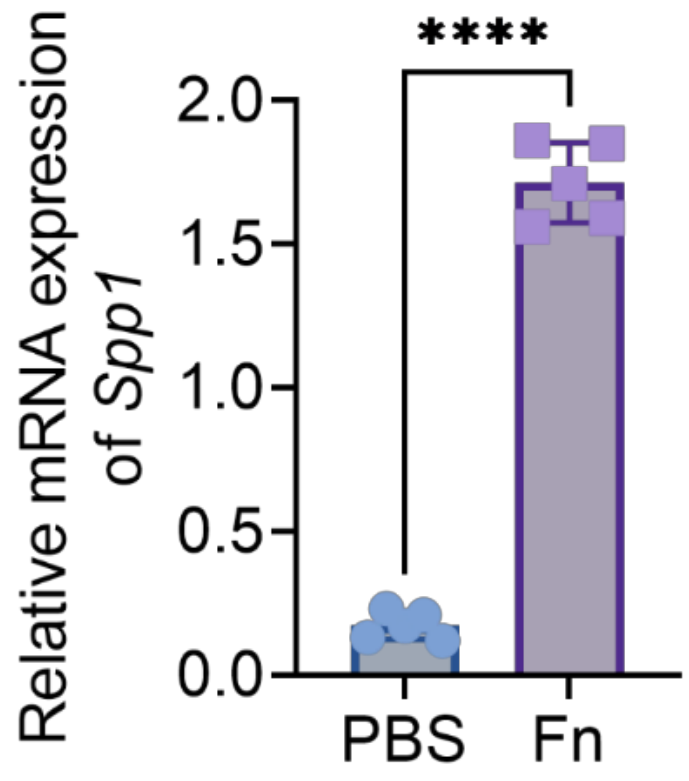

Figure S18 C57BL/6 mice bearing subcutaneous mouse-derived ESCC tumors received PBS and Fn (1×10^8 CFU, multipoint intratumoral, twice weekly × 2 weeks). RT-qPCR was performed to determine SPP1 mRNA level.

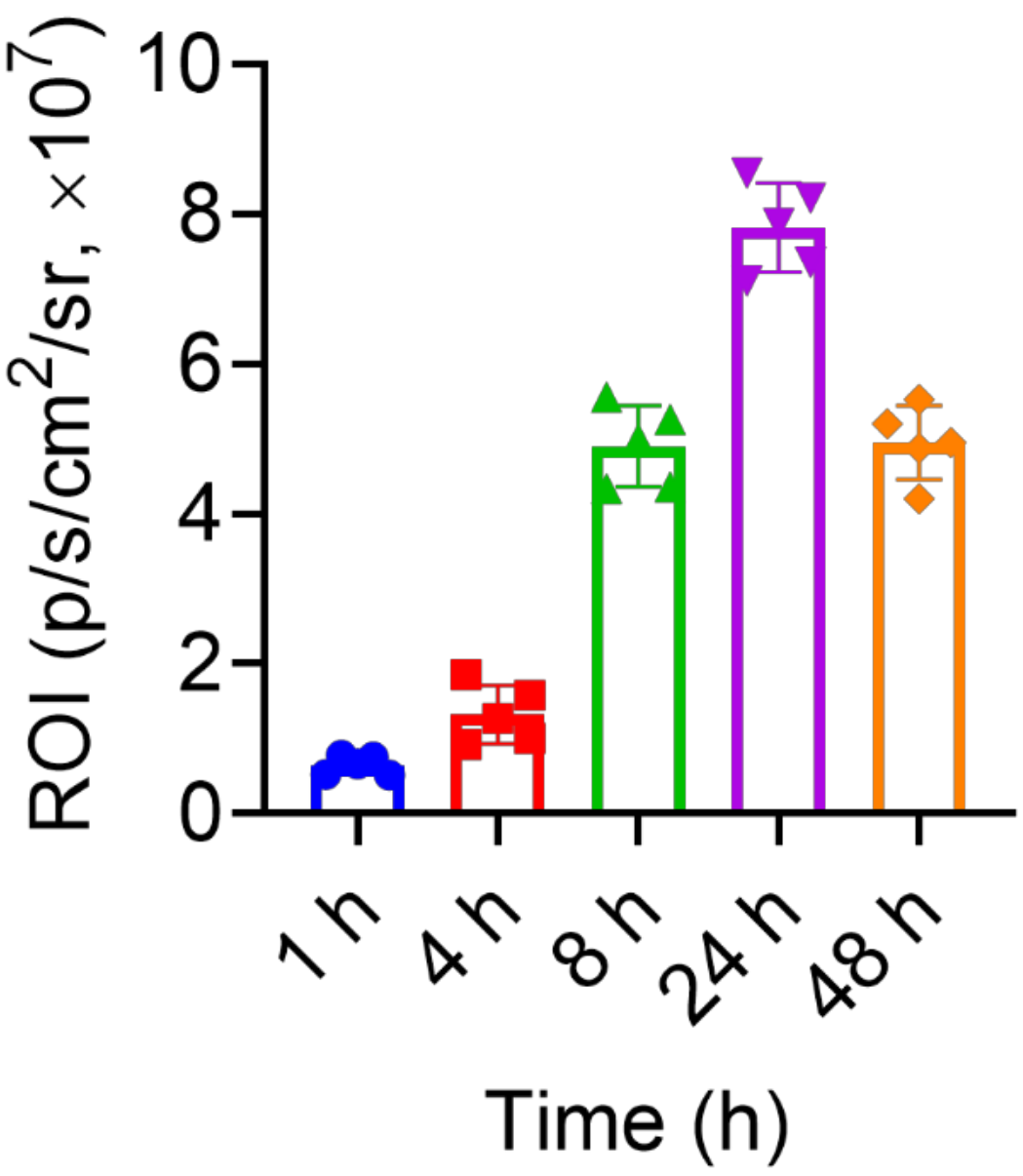

Figure S19 C57BL/6 mice bearing subcutaneous mouse-derived ESCC tumors received Fn (1×10^8 CFU, multipoint intratumoral, twice weekly × 2 weeks). When tumors reached ≈100 mm^3 (Day 0). In vivo imaging and quantitative statistical analysis of the fluorescence intensity at the tumor site at different time points after intravenous injection of Cy5-labeled NC-TNP^M.

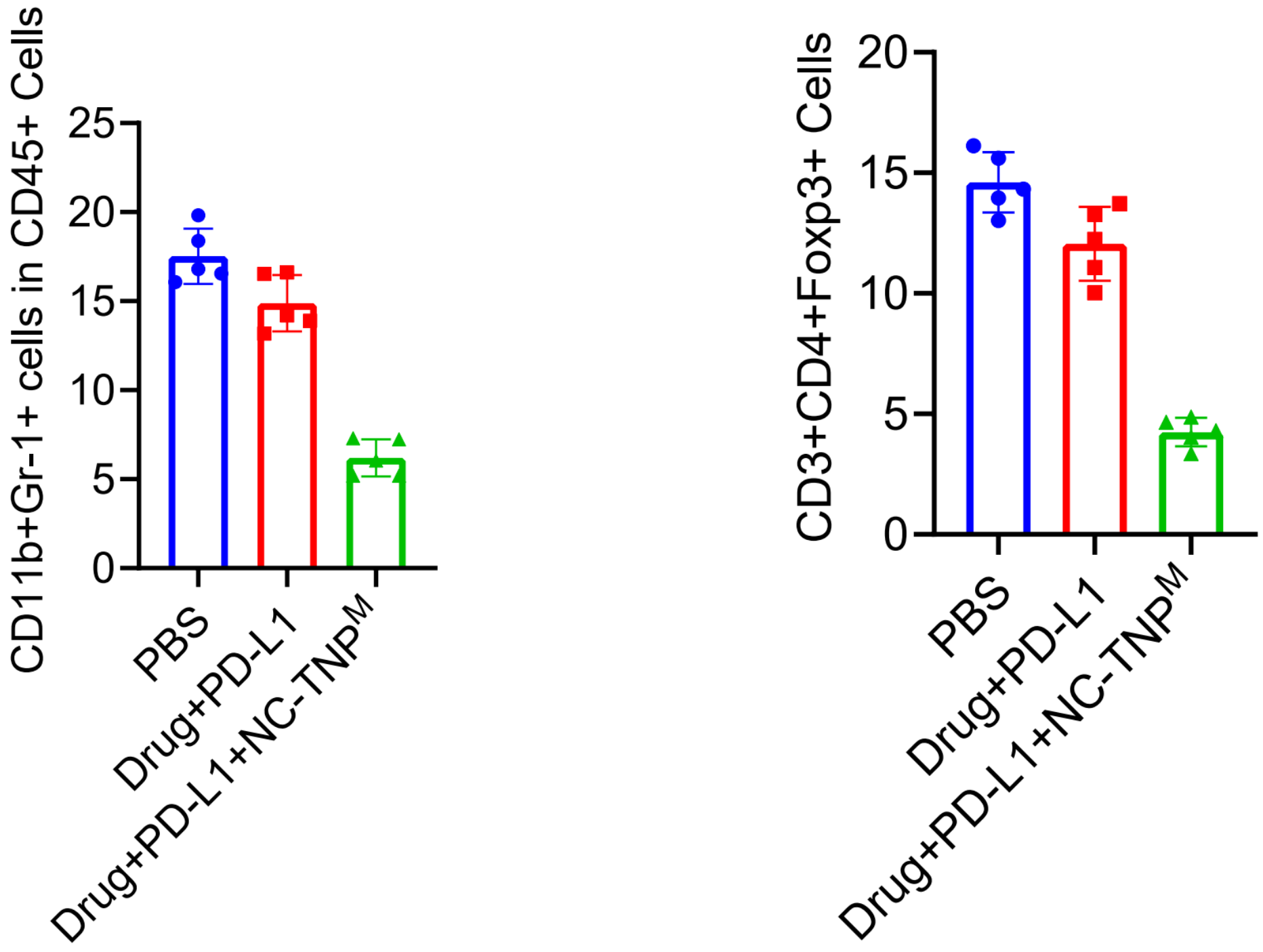

Figure S20 After excision, tumor tissues were digested with collagenase to generate a single-cell suspension, which was then analyzed by flow cytometry to determine the proportions of CD11b+Gr1+ cells and CD3+CD4+Foxp3+ cells (mean ± SD; n=5).

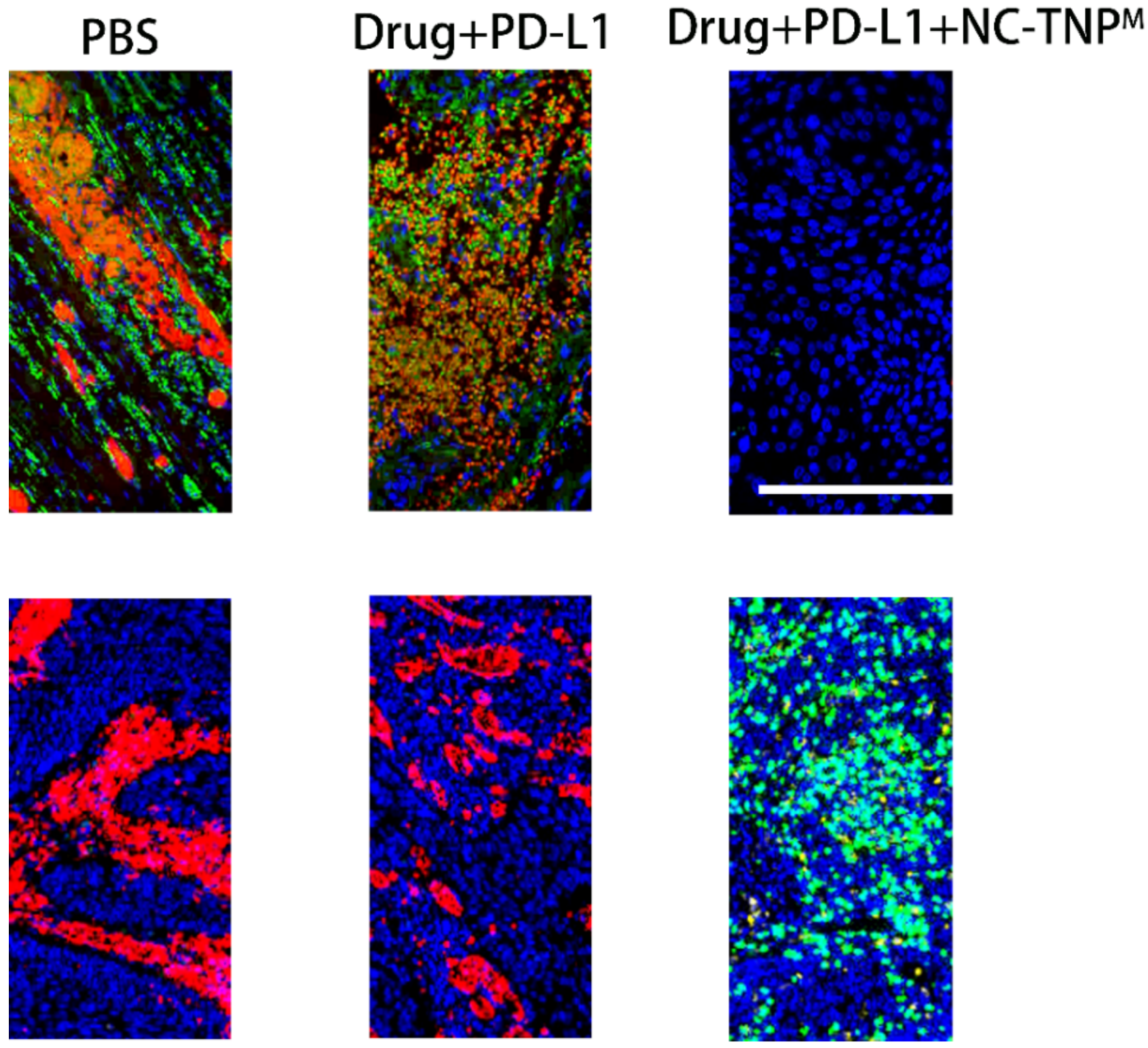

Figure S21 Zoom in on a specific area of Figure 5F, scale bar: 200 μm.
